# Generating a Metal-responsive Transcriptional Regulator to Test What Confers Metal Sensing in Cells*[Fn FN2]

**DOI:** 10.1074/jbc.M115.663427

**Published:** 2015-06-24

**Authors:** Deenah Osman, Cecilia Piergentili, Junjun Chen, Buddhapriya Chakrabarti, Andrew W. Foster, Elena Lurie-Luke, Thomas G. Huggins, Nigel J. Robinson

**Affiliations:** From the ‡ School of Biological and Biomedical Sciences and Department of Chemistry, Durham University, Durham DH1 3LE, United Kingdom,; § Procter and Gamble, Mason Business Centre, Cincinnati, Ohio 45040, and; ¶ Life Sciences Open Innovation, London Innovation Centre, Procter and Gamble Technical Centres, Ltd., Egham TW20 9NW, United Kingdom

**Keywords:** copper, metal, metal homeostasis, metalloprotein, Salmonella enterica, zinc, FrmR, RcnR-CsoR, cobalt, metal-sensor

## Abstract

FrmR from *Salmonella enterica* serovar *typhimurium* (a CsoR/RcnR-like transcriptional de-repressor) is shown to repress the *frmRA* operator-promoter, and repression is alleviated by formaldehyde but not manganese, iron, cobalt, nickel, copper, or Zn(II) within cells. In contrast, repression by a mutant FrmRE64H (which gains an RcnR metal ligand) is alleviated by cobalt and Zn(II). Unexpectedly, FrmR was found to already bind Co(II), Zn(II), and Cu(I), and moreover metals, as well as formaldehyde, trigger an allosteric response that weakens DNA affinity. However, the sensory metal sites of the cells' endogenous metal sensors (RcnR, ZntR, Zur, and CueR) are all tighter than FrmR for their cognate metals. Furthermore, the endogenous metal sensors are shown to out-compete FrmR. The metal-sensing FrmRE64H mutant has tighter metal affinities than FrmR by approximately 1 order of magnitude. Gain of cobalt sensing by FrmRE64H remains enigmatic because the cobalt affinity of FrmRE64H is substantially weaker than that of the endogenous cobalt sensor. Cobalt sensing requires glutathione, which may assist cobalt access, conferring a kinetic advantage. For Zn(II), the metal affinity of FrmRE64H approaches the metal affinities of cognate Zn(II) sensors. Counter-intuitively, the allosteric coupling free energy for Zn(II) is smaller in metal-sensing FrmRE64H compared with nonsensing FrmR. By determining the copies of FrmR and FrmRE64H tetramers per cell, then estimating promoter occupancy as a function of intracellular Zn(II) concentration, we show how a modest tightening of Zn(II) affinity, plus weakened DNA affinity of the apoprotein, conspires to make the relative properties of FrmRE64H (compared with ZntR and Zur) sufficient to sense Zn(II) inside cells.

## Teaser

**Background:** Metal-specific transcription has been correlated with the relative properties of a cells' set of metal sensors.

**Results:** A one-residue substitution enabled a DNA-binding formaldehyde sensor to detect Zn(II) and cobalt.

**Conclusion:** Weaker DNA affinity combined with tighter Zn(II) affinity enabled Zn(II) sensing with a smaller coupling free energy.

**Significance:** Relative affinity determined the best sensor in the set for Zn(II) but not for cobalt.

## Introduction

Metal-sensing and DNA-binding transcriptional regulators are central to the machinery that optimizes buffered metal concentrations inside cells to enable correct protein metallation (1, 2). In general, the tighter the *K*_metal_ of a metal sensor, the lower the [buffered metal] (1). Fresh experimental approaches are needed to test hypotheses about the mechanisms determining which metal(s) each sensor detects. Uncertainty also remains about the nature of the exchangeable pools of different metals, including the major ligands and the precise buffered metal concentrations, and how these vary under different environmental conditions or between organisms.

Metal sensors tend to bind divalent metals with an order of affinity that matches the Irving Williams series, regardless of which metal(s) they detect in a cell (1–3). This raises questions about how a sub-set of sensors can detect the weaker binding metals *in vivo* (4–6). One facet of the solution is that the kinetics of access to different metals can vary from sensor to sensor, for example due to interactions with specific donor molecules, including metallochaperones (1, 6–8). Another part of the solution is that the allosteric mechanism connecting metal binding to DNA binding can be metal-selective (9–12). Thus, a weaker binding metal can nonetheless be more effective at triggering the conformational changes that alter gene expression (10, 13). For metal-dependent de-repressors and co-repressors the coupling free energy, Δ*G*_C_^metal-sensor·DNA^, is typically larger for more effective metals (9). Unexpectedly, here we see how a metal can also become effective without increasing Δ*G*_C_^metal-sensor·DNA^, if *K*_DNA_ of the apo-form of a de-repressor is suitably weakened, to confer two mechanistic advantages in favor of Zn(II)- detection. Contrary to general dogma, here Δ*G*_C_^Zn(II)-sensor·DNA^ is smaller in the Zn(II)-sensing mutant relative to the nonsensing wild type protein.

**FIGURE 1.**
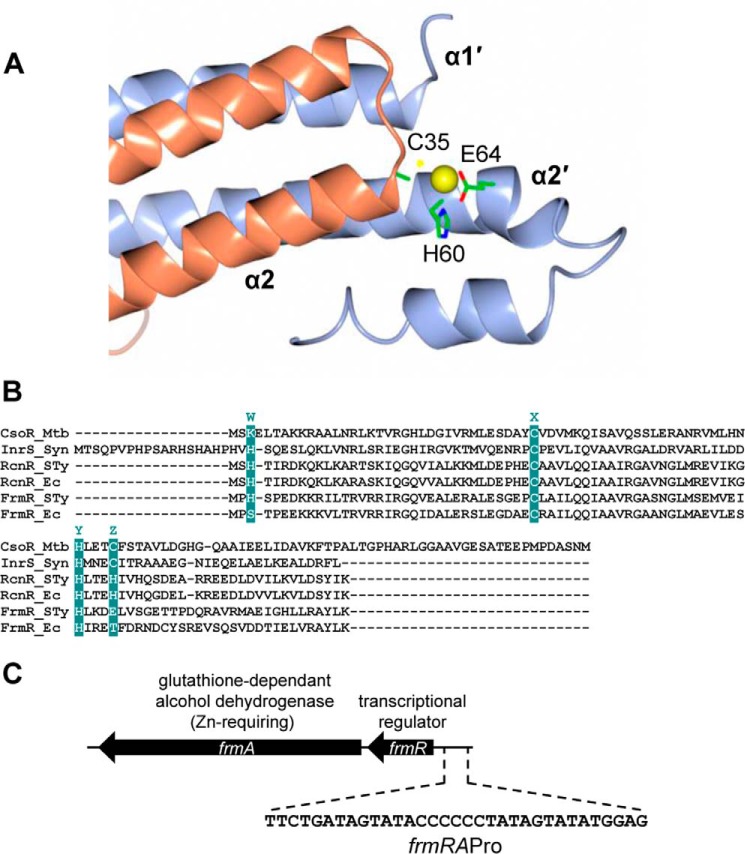
**FrmR candidate metal-binding ligands and genetic organization.** *A*, dimeric representation of *M. tuberculosis* Cu(I)-CsoR (Protein Data Band code 2HH7) with replacement of residue 65 (cysteine) with glutamate as found at analogous residue 64 in FrmR. Side chains of putative FrmR-metal ligands (with FrmR numbering) are shown with metal ion depicted as a *yellow sphere. B,* alignment of *M. tuberculosis* CsoR (CsoR_Mtb), *Synechocystis* PCC 6803 InrS (InrS_Syn), *Salmonella* RcnR (RcnR_STy), *E. coli* RcnR (RcnR_Ec), *Salmonella* FrmR (FrmR_STy), and *E. coli* FrmR (FrmR_Ec). The residues at positions of the WXYZ fingerprint are highlighted (15, 17). *C*, schematic representation of the *frmRA* operon (to scale) from *Salmonella* indicating the *frmRA* promoter, which includes a candidate FrmR-binding site and forms one strand of *frmRA*Pro.

In the course of a collaborative program to characterize the complement of metal sensors from *Salmonella enterica* serovar *typhimurium* strain SL1344 (hereafter referred to as *Salmonella*), we identified two genes encoding proteins with sequence similarity to members of the CsoR/RcnR family of DNA-binding and metal-responsive transcriptional de-repressors (14–17). These are now shown to be *Salmonella* homologues of RcnR and FrmR. RcnR in *Escherichia coli* responds to cobalt and nickel, whereas CsoR, first discovered in *Mycobacterium tuberculosis*, responds to Cu(I) (15–17). Related metal sensors characterized from other bacteria detect the same metals (18–27). Additionally, two homologues have been identified that respond to effectors other than metals, namely CstR from *Staphylococcus aureus,* which detects persulfide, plus *E. coli* FrmR (28–30). CsoR forms a three helix bundle that assembles into tetramers (15). The sensory Cu(I) site exploits a conserved Cys-thiolate from the N-terminal end of helix α2 of one subunit in combination with an H*XXX*C motif from within helix α2′ of a second subunit (Fig. 1*A*) (15). Three ligands in similar locations (with H*XXX*C replaced by H*XXX*H), along with additional ones from the N-terminal region of helix α1′, are recruited to the sensory metal site of RcnR (Fig. 1*B*) (17, 31, 32). A single residue variant of *E. coli* RcnR (H3E) also responds to Zn(II) (31).

In a global screen to discover the consequences of the read-through of amber stop codons, *E. coli* FrmR (which has such a stop) emerged as the transcriptional repressor of the *frmRAB* operon (30). FrmA has formaldehyde dehydrogenase activity, and the operon was subsequently shown to respond to exogenous formaldehyde (30, 33). This operon is de-repressed during anaerobic respiration using trimethylamine *N*-oxide as the terminal electron acceptor where endogenous formaldehyde is generated as a by-product of trimethylamine *N*-oxide demethylation (34). CO-releasing molecules and chloride treatments also trigger expression of the *frm* operon (35, 36). There are no published studies of the *Salmonella* FrmR homologue. At least two potential metal ligands are retained in *Salmonella* FrmR, namely Cys at the N terminus of helix α2 but H*XXX*E (rather than H*XXX*H of paralogous *Salmonella* RcnR) at helix α2′ (Fig. 1, *A* and *B*). Despite sequence similarity between FrmR and other CsoR/RcnR family members, whether or not (any) FrmRs de-repress gene expression in response to metals remains untested.

Recent studies have shown that relative affinity, relative allostery, and relative access determine the ability of metal sensors to respond selectively *in vivo* (1). This is exemplified by comparing metal affinities (*K*_metal_) and metal-responsive allostery (Δ*G*_C_^metal-sensor·DNA^) among multiple metal sensors, and for multiple metals in *Synechocystis* PCC 6803 (1, 6, 11, 18). Thus, InrS responds to nickel *in vivo* and has a *K*_Ni(II)_ that is substantially tighter than *K*_Ni(II)_ of cobalt-sensing CoaR and Zn(II)-sensing ZiaR or Zur (a representative from each family of metal sensors that is present in this organism) (18). Provided the distribution of Ni(II) follows thermodynamic equilibrium predictions, as the [Ni(II)] rises, InrS will be the first to respond, de-repressing expression of *nrsD* (encoding a Ni(II)-efflux protein) and preventing [Ni(II)] from approaching *K*_Ni(II)_ of the other sensors (18). For Zn(II), *K*_Zn(II)_ of nickel-sensing InrS is similar to Zn(II)-sensing ZiaR, but crucially the allosteric mechanism of ZiaR is more responsive to Zn(II) compared with InrS (Δ*G*_C_^Zn(II)-ZiaR·DNA^ > Δ*G*_C_^Zn(II)-InrS·DNA^) (11). Thus ZiaR will require a lower fractional Zn(II) occupancy than InrS to de-repress its target gene *ziaA* (encoding a Zn(II)-efflux ATPase). In this manner, ZiaR can prevent [Zn(II)] from exceeding the threshold where occupancy of DNA by InrS becomes sufficiently low for aberrant expression of *nrsD* to occur (11). In contrast, cobalt sensing does not correlate with relative affinity, and CoaR has the weakest *K*_Co(II)_ in the set of sensors (6). There is evidence that the cobalt effector may be preferentially channelled to CoaR, and thus relative access has been invoked as the explanation for selective detection of cobalt (6). In summary, it is hypothesized that the sensor, which is triggered by a metal, is simply the most responsive within a cells' set of sensors, based upon relative affinity, relative allostery, and relative access (1). This hypothesis is now tested via a mutation conferring gain-of-metal sensing.

Here, the Co(II), Zn(II)-, and Cu(I)-binding affinities of *Salmonella* FrmR are determined and compared with equivalent data for the cognate sensors of these metals, namely *Salmonella* homologues of RcnR, Zn(II)-sensing ZntR and Zur, and Cu(I)-sensing CueR. FrmR is found not to sense metals within cells, yet an E64H substitution (creating a *Salmonella* RcnR-like helix α2′ H*XXX*H motif) gains responsiveness to cobalt and Zn(II) *in vivo*. By comparing the biochemical properties of *Salmonella* FrmR with FrmRE64H, and then relating these parameters to endogenous sensors for cobalt, Zn(II), and Cu(I), the relative properties that, in combination, enable metal sensing are identified.

## Experimental Procedures

### 

#### 

##### Bacterial Strains and DNA Manipulations

*S. enterica* sv. *typhimurium* strain SL1344 was used as wild type, and strain LB5010a was used as a restriction-deficient modification-proficient host for DNA manipulations. Both were a gift from J. S. Cavet (University of Manchester). *E. coli* strain DH5α was used for routine cloning. Bacteria were cultured with shaking at 37 °C in Luria-Bertani (LB) medium or M9 minimal medium (37), supplemented with thiamine (0.001% w/v) and l-histidine (20 μg ml^−1^). Carbenicillin (100 μg ml^−1^), kanamycin (50 μg ml^−1^), and/or chloramphenicol (10 μg ml^−1^) were added where appropriate. Cells were transformed to antibiotic resistance as described (37, 38). All generated plasmid constructs were checked by sequence analysis. Primers are listed in supplemental Table S1.

##### Generation of Salmonella Deletion Mutants

Deletion derivatives of strain LB5010a were obtained using the λ Red method (38) using plasmid pKD3 and primers 1 and 2 for *frmR* or primers 3 and 4 for *gshA*. Mutagenesis was performed using strain LB5010a and selection of mutants achieved using LB medium supplemented with chloramphenicol. Mutations were subsequently moved to SL1344 or derivatives using P22 phage transduction and validated by PCR using primers 5 and 6 for *frmR* or primers 7 and 8 for *gshA*. The antibiotic resistance cassette from the Δ*frmR*::*cat* locus was removed using the helper plasmid pCP20 carrying the FLP recombinase.

##### Generation of Promoter-lacZ Fusion Constructs and β-Galactosidase Assays

P*_frmRA_* or P*_frmRA_-frmR* was amplified from SL1344 genomic DNA using primer 9 and either primer 10 (for P*_frmRA_*) or 11 (for P*_frmRA_-frmR*) and ligated into pGEM-T. Site-directed mutagenesis to generate P*_frmRA_-frmRE64H* and P*_frmRA_-frmR*^DOWN^ was conducted via the QuikChange® protocol (Stratagene) using pGEM-P*_frmRA_-frmR* as template and primers 12–23. Codon optimization of the *frmRE64H* coding region to generate P*_frmRA_-frmRE64H*^UP^ (supplemental Table S2) was achieved using GeneArt Gene Synthesis (Life Technologies, Inc.) and optimization for *Salmonella typhimurium*. The *rcnR-*P*_rcnA_* region was amplified from SL1344 genomic DNA using primers 24 and 25. Digested fragments were cloned into the SmaI/BamHI site of pRS415 (39). P*_zntA_* cloned into pRS415 was provided by J. S. Cavet (University of Manchester). The resulting constructs were introduced into strain LB5010a prior to strain SL1344. β-Galactosidase assays were performed as described (40) in triplicate on at least three separate occasions. Overnight cultures were grown in M9 minimal medium, diluted 1:50 in fresh medium supplemented with maximum noninhibitory concentrations (MNIC^3^; defined as the maximum concentration which inhibited growth by ∼ 10%) of metals, formaldehyde, EDTA, or *N,N,N′,N′*-tetrakis(2-pyridylmethyl)ethylenediamine (TPEN), and grown to mid-logarithmic phase prior to assays. For time course experiments, cells were grown to early logarithmic phase and statically cooled to 25 °C for 20 min followed by a 2-h incubation in the presence of metal or formaldehyde. The metal salts used were MnCl_2_, C_6_H_5_FeO_7_, CoCl_2_, NiSO_4_, CuSO_4_, and ZnSO_4_, and the concentrations were verified by ICP-MS. MNICs under these growth conditions were 200 μm MnCl_2_, 5 μm CoCl_2_, 20 μm NiSO_4_, 25 μm CuSO_4_, 50 μm ZnSO_4_, 50 μm formaldehyde, 25 μm EDTA, and 0.25 μm TPEN, with the exception that 1 μm CoCl_2_ was found to be the MNIC for cells expressing *rcnR-*P*_rcnA_*. Addition of C_6_H_5_FeO_7_ improved growth, and 5 μm was used throughout.

##### Protein Expression and Purification

The *frmR*, *zntR*, *zur*, and *rcnR* coding regions were amplified from SL1344 genomic DNA using primers 26–33 and ligated directly into pET29a (Novagen) (or via pGEM-T) using the NdeI/BamHI site for *frmR*, *zntR,* and *rcnR* or NdeI/EcoRI site for *zur*. Site-directed mutagenesis was conducted as described above using template pETfrmR and primers 34 and 35 to generate pETfrmRE64H or template pETzntRLT2 and primers 36 and 37 to generate pETzntR. Proteins were expressed in exponentially growing *E. coli* BL21(DE3) for 3 h at 37 °C using 0.2 mm isopropyl 1-thio-β-d-galactopyranoside. The medium was supplemented with 50 μm ZnSO_4_ for Zur (to promote metallation of the structural zinc site). Harvested BL21(DE3) pETfrmR or pETfrmRE64H cells were resuspended in Buffer A (300 mm NaCl, 5 mm imidazole, 5 mm DTT, 50 mm sodium phosphate, pH 7.4) with the addition of 1 mm PMSF and, following sonication and clarification, were applied to an equilibrated 5-ml HisTrap FF column (GE Healthcare), washed in the same buffer, and eluted in a single step using Buffer A with 300 mm imidazole. BL21(DE3) pETzntR or pETzur cells were treated in the same way, except using Buffer A with 20 mm sodium phosphate, pH 7.4, and elution with 100 mm imidazole. BL21(DE3) pETrcnR cells were resuspended in Buffer B (300 mm NaCl, 10 mm EDTA, 10 mm DTT, 10 mm HEPES, pH 7.0) with the addition of 1 mm PMSF and (post-sonication and clarification) applied to an equilibrated 5-ml HiTrap heparin column (GE Healthcare), washed in the same buffer, and eluted in a single step using Buffer B with 800 mm NaCl. Proteins were further purified by size-exclusion chromatography (HiLoad 26/60 Superdex 75, GE Healthcare) equilibrated in 300 mm NaCl, 10 mm DTT, 10 mm EDTA, 10 mm HEPES pH 7.8 for FrmR and FrmRE64H; 50 mm NaCl, 5 mm DTT, 1 mm EDTA, 10 mm HEPES, pH 7.0, for ZntR; 300 mm NaCl, 5 mm DTT, 1 mm EDTA, 10 mm HEPES pH 7.8 for Zur; and Buffer B for RcnR. FrmR and FrmRE64H were diluted to 100 mm NaCl, 10 mm DTT, 10 mm EDTA, 10 mm HEPES, pH 7.8, applied to an equilibrated 1-ml HiTrap heparin column (GE Healthcare), and washed with 10 column volumes of the same buffer. ZntR and Zur were treated the same way, except using 5 mm NaCl, 1 mm EDTA, 5 mm DTT, 10 mm HEPES, pH 7.0, for ZntR, and 100 mm NaCl, 5 mm DTT, 1 mm EDTA, 10 mm HEPES pH 7.8 for Zur. FrmR, FrmRE64H, ZntR, and Zur were eluted in a single step using respective binding buffers plus 500 mm NaCl. RcnR was diluted to 100 mm NaCl, 10 mm EDTA, 10 mm DTT, 10 mm HEPES, pH 7.0, and applied to an equilibrated 5-ml HiTrap SP column (GE Healthcare), washed in the same buffer plus 200 mm NaCl, and eluted in 300 mm NaCl. CueR was expressed and purified as described previously (41). Anaerobic protein stocks were prepared by applying purified protein to a pre-equilibrated 1-ml HiTrap heparin column (diluting FrmR, FrmRE64H, ZntR, and Zur as described above and without dilution of RcnR). The protein-loaded column was moved into an anaerobic chamber, washed with >10 column volumes of Chelex-treated, N_2_-purged 80 mm KCl, 20 mm NaCl, 10 mm HEPES, pH 7.0, for FrmR, FrmRE64H, and Zur; 4 mm KCl, 1 mm NaCl, 10 mm HEPES, pH 7.0, for ZntR; or 240 mm KCl, 60 mm NaCl, 10 mm HEPES, pH 7.0, for RcnR. Proteins were eluted in a single step using 400 mm KCl, 100 mm NaCl, 10 mm HEPES, pH 7.0, for FrmR, FrmRE64H, Zur, and ZntR, or 800 mm KCl, 200 mm NaCl, 10 mm HEPES, pH 7.0, for RcnR. Proteins were quantified by measurement of *A*_280 nm_ and using experimentally determined extinction coefficients obtained via quantitative amino acid analysis (Abingdon Health Laboratory Services). These were 1951 m^−1^ cm^−1^ for FrmR and FrmRE64H, and 11,505, 4823, 2422, and 5136 m^−1^ cm^−1^ for ZntR, Zur, RcnR, and CueR, respectively. It was noted that the absorbance spectra of FrmRE64H differed from FrmR (by exhibiting a shoulder at ∼300 nm), except in two early preparations, and these were not used further. Reduced thiol and metal content were assayed as described previously (18), and all anaerobic protein samples (maintained in an anaerobic chamber) were ≥90% reduced and ≥95% metal-free, with the exception of Zur which contained ∼1 m eq of Zn(II) (per monomer) as purified. All *in vitro* experiments were carried out under anaerobic conditions using Chelex-treated and N_2_-purged buffers as described previously (18).

##### UV-visible Absorption Spectroscopy

Experiments were carried out in 100 mm NaCl, 400 mm KCl, and 10 mm HEPES, pH 7.0, for FrmR, FrmRE64H, ZntR, Zur, and CueR, with inclusion of 5% (v/v) glycerol for RcnR. Concentrations of metal stocks (CoCl_2_, NiCl_2_, CuCl, and ZnCl_2_) were verified by ICP-MS. CuCl was prepared as described previously and confirmed to be >95% Cu(I) by titration against bathocuproine sulfonate (BCS) (42). CoCl_2_, NiCl_2_, or CuCl (>95% Cu(I)) were titrated into protein, or ZnCl_2_ was titrated into protein pre-equilibrated with CoCl_2_, and the absorbance spectra were recorded at equilibrium using a λ35 UV-visible spectrophotometer (PerkinElmer Life Sciences). Precipitation of ZntR was observed with further ZnCl_2_ additions to Co(II)-ZntR than those shown.

##### Protein-Metal Migration by Size-exclusion Chromatography

FrmR, FrmRE64H, or Zur were incubated (60 min) with an excess of ZnCl_2_, CuCl (>95% Cu(I)) or EDTA (as stated) in 100 mm NaCl, 400 mm KCl, and 10 mm HEPES, pH 7.0, and an aliquot (0.5 ml) was resolved by size-exclusion chromatography (PD-10 Sephadex G25, GE Healthcare) in the same buffer conditions. Fractions (0.5 ml) were analyzed for metal by ICP-MS and protein by the Bradford assay using known concentrations of FrmR, FrmRE64H, or Zur as standards. Failure to recover all of the copper during experimentation with FrmR or FrmRE64H suggests (at least) some competition from and copper binding by the Sephadex matrix.

##### Protein-Chelator-Zn(II) Competitions

Experiments were carried out in 100 mm NaCl, 400 mm KCl, and 10 mm HEPES, pH 7.0, as described previously (11). ZnCl_2_ was titrated into a mixed solution of protein and mag fura-2 or protein and quin-2, and absorbance was recorded at equilibrium at 366 nm (mag fura-2) and 261 or 265 nm (quin-2). Data were fit to the models described in the figure legends and Table 1 footnotes using Dynafit (43) to determine Zn(II) binding constants. Mag fura-2 and quin-2 were quantified using extinction coefficients ϵ_369 nm_ = 22,000 m^−1^ cm^−1^ (44) and ϵ_261 nm_ = 37,000 m^−1^ cm^−1^ (45), respectively. *K*_Zn(II)_ = 2 × 10^−8^ m for mag-fura-2 at pH 7.0 (46), and *K*_Zn(II)_ = 3.7 × 10^−12^ m at pH 7.0 for quin-2 (45).

##### Protein-Chelator-Co(II) Competitions

CoCl_2_ was titrated into a mixed solution of protein and fura-2 or protein and BisTris in 100 mm NaCl, 400 mm KCl, and 10 mm HEPES, pH 7.0, with the addition of 5% (v/v) glycerol for experiments with RcnR. For competition with fura-2, fluorescence emission was recorded at equilibrium at 510 nm (λ_ex_ = 360 nm; *T* = 20 °C) using a Cary Eclipse fluorescence spectrophotometer (Agilent Technologies), as described previously (6). Fura-2 was quantified using the extinction coefficient ϵ_363 nm_ = 28,000 m^−1^ cm^−1^ (6). For competition with BisTris, absorbance spectra were recorded at equilibrium. Data were fit to the models described in the figure legends and Table 1 footnotes using Dynafit to determine Co(II)-binding constants (43). *K*_Co(II)_ = 8.64 × 10^−9^ m for fura-2 at pH 7.0 (47), and *K*_Co(II)_ = 2.26 × 10^−2^ m at pH 7.0 for BisTris using the absolute formation constant for Co(II)-BisTris and Schwarzenbach's α-coefficient method (48).

##### Protein-Chelator-Cu(I) Competitions

Experiments were carried out in 100 mm NaCl, 400 mm KCl, and 10 mm HEPES, pH 7.0. CuCl (> 95% Cu(I)) was titrated into a mixed solution of protein and BCA, and the absorbance at 562 nm was recorded at equilibrium. Data were fit to the models described in the figure legends and Table 1 footnotes using Dynafit to determine Cu(I)-binding constants (43). β_2Cu(I)_ = 1.58 × 10^17^ m^−2^ at pH 7.0 for BCA (48). For BCS, the absorbance at 483 nm was recorded following titration with CuCl (to generate a calibration curve) or following preincubation of BCS with CuCl (10 min) and addition of CueR. The absorbance at 483 nm was monitored to equilibrium. *K*_Cu(I)_ of the tightest site of CueR was calculated using Equation 1 (48),

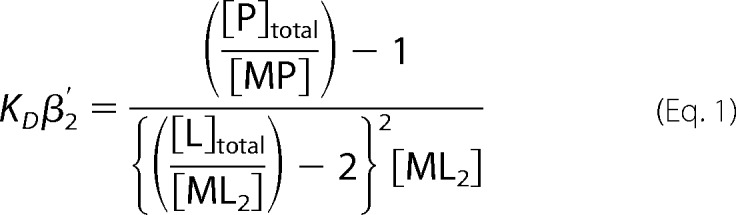
 CueR is expected to be a dimer with two metal-binding sites (49) that bind Cu(I) with negative cooperativity (41); therefore, the concentration of the tightest metal-binding site, [P]_total_, was taken as [CueR monomer] × 0.5. [L]_total_ is the total [BCS]. The absorbance at 483 nm at the end point of competition between CueR and BCS was used to calculate [Cu(I)BCS_2_] from the slope of the calibration curve. Assuming all Cu(I) is bound to either CueR or BCS, the concentration of Cu(I)-CueR [MP], was determined by subtracting [ML_2_] from total metal [M_total_] used in the competition. β′_2Cu(I)_ = 6.01 × 10^19^ m^−2^ at pH 7.0 for BCS using the absolute formation constant of Cu(I)-BCS_2_ and Schwarzenbach's α-coefficient method (48).

##### Fluorescence Spectroscopy

Experiments were carried out in 100 mm NaCl, 400 mm KCl, 10 mm HEPES, pH 7.0. ZnCl_2_ was titrated into ZntR, and fluorescence emission spectra (λ_ex_ = 280 nm, λ_em_ = 303 nm, and *T* = 20 °C) were recorded at equilibrium using a Cary Eclipse fluorescence spectrophotometer. Precipitation was observed with addition of more than 1.1 m eq ZnCl_2_.

##### Interprotein Metal Exchange

For competition of FrmR with CueR or ZntR, FrmR (40 μm, monomer) was equilibrated with 10 μm CuCl (>95% Cu(I)) or ZnCl_2_, in 100 mm NaCl, 400 mm KCl, 10 mm HEPES, pH 7.0, before addition of either 20 μm (monomer) CueR or ZntR, respectively. Protein mixtures (1 ml) were diluted to 20 mm NaCl, 80 mm KCl, 10 mm HEPES, pH 7.0, and applied to a heparin affinity chromatography column. FrmR and CueR were differentially eluted in 60 mm NaCl, 240 mm KCl, 10 mm HEPES, pH 7.0. ZntR does not bind the column, and FrmR was eluted with 100 mm NaCl, 400 mm KCl, 10 mm HEPES, pH 7.0. Fractions (1 ml) were assayed for metal by ICP-MS and protein by SDS-PAGE. For competition of FrmR with RcnR, apo-subtracted difference spectra were taken at equilibrium of FrmR or RcnR incubated with 9.9 μm CoCl_2_, or after addition of RcnR to Co(II)-FrmR (using the same concentrations as control). Buffer conditions were 100 mm NaCl, 400 mm KCl, 10 mm HEPES, pH 7.0.

##### Protein Quantification by Liquid Chromatography-Tandem Mass Spectrometry

Cellular lysates were prepared from logarithmic cultures grown in M9 minimal medium. Cell number was determined by enumeration on LB agar plates. Harvested cells were resuspended in 40 mm NaCl, 160 mm KCl, 10 mm EDTA, 10 mm DTT, 10 mm HEPES, pH 7.8, with addition of protease inhibitor mixture (Sigma), and post-sonication, the soluble cell lysate was syringe-filtered (0.45-μm pore size), snap-frozen in liquid N_2_, stored at −80 °C, and thawed on ice before use. Total protein was determined by the Bradford assay, using BSA as a standard. Purified stocks of FrmR or FrmRE64H were quantified by amino acid analysis (Proteomics Core Facility, University of California), stored at −80 °C, and thawed on ice before dilution in PBS to 0.6 mg ml^−1^. For standard curves, proteins were further diluted in soluble cell lysates from Δ*frmR* cells to generate standard curve concentrations of 5, 10, 50, 250, 425, and 500 ng of 100 μl^−1^ (which defined the limits for quantification). Aliquots were stored at −80 °C. Working internal standards were prepared by dilution of labeled peptides ([^13^C_6_,^15^N_4_]arginine residues) GQVEALER[^13^C_6_,^15^N_4_], DELVSGETTPDQR[^13^C_6_,^15^N_4_], and DHLVSGETTPDQR[^13^C_6_,^15^N_4_] (Thermo Fisher) in 15% (v/v) acetonitrile with 0.1% (v/v) formic acid solution to obtain final concentrations of 313 fmol μl^−1^ of each peptide. For experimental samples (soluble lysates from Δ*frmR* cells containing the P*_frmRA_-frmR* construct and variants) and standard curve samples, 100 μl was precipitated using 300 μl of methanol (mixing at 2000 rpm for 1 min) before centrifugation (900 × *g*, 5 min, room temperature). Pellets were suspended in 400 μl of 200 mm NH_4_HCO_3_ in 10% (v/v) methanol (mixing at 2000 rpm for 10 min) and 10 μl internal standard added. Pellet digestion was performed with 10 μl of trypsin (14 mg ml^−1^) and mixing (1000 rpm, 37 °C, 16 h) and stopped with 10 μl of 15% (v/v) formic acid. The digested samples were centrifuged (6000 × *g* for 5 min at room temperature) to remove particulate material. Solvent was removed from clarified supernatants (50–100 μl) using a centrifugal evaporator (Thermo Scientific SpeedVac system). Samples were separated by gradient elution at 0.3 ml min^−1^ using a Zorbax Eclipse Plus C18 column (2.1 × 150 mm, 3.5-μm particles; Agilent Technologies) at 30 °C. Mobile phase A and B consisted of 0.1% (v/v) formic acid in water and 0.1% (v/v) formic acid in acetonitrile, respectively. Aliquots (20 μl) were applied to a 6500 triple quadrupole mass spectrometer (AB Sciex) operating in positive ionization mode. Acquisition methods used the following parameters: 5500 V ion spray voltage; 25 p.s.i. curtain gas; 60 p.s.i. source gas; 550 °C interface heating temperature; 40 V declustering potential; 26 V collision energy; and 27 V collision cell exit potential. Scheduled multiple reaction monitoring was carried out with a 90-s multiple reaction monitoring detection window and 1.00-s target scan time. A quadratic 1/*x*^2^ weighted regression model was used to perform standard calibration. The coefficient of determination (*R*^2^) was >0.990 for GQVEALER in all validation runs.

##### Determining Intracellular [Glutathione]

Intracellular glutathione was measured using a glutathione assay kit (Sigma) according to the manufacturer's instructions. Cellular lysates were prepared from overnight cultures grown in M9 minimal medium, diluted 1:50 in fresh medium, and grown to early logarithmic phase, statically cooled to 25 °C for 20 min, followed by 30-min incubation in the absence or presence of MNIC ZnSO_4_. Viable cells were enumerated on LB agar, and [glutathione] was calculated using a cell volume of 1 fl.

##### Fluorescence Anisotropy

Complementary single-stranded oligonucleotides 38 (hexachlorofluorescein-labeled) and 39 (containing the identified FrmR-binding site and flanking nucleotides, Fig. 1*C*) were annealed by heating 10 or 200 μm of each strand in 10 mm HEPES, pH 7.0, 150 mm NaCl to 95 °C, and cooled to room temperature overnight. For protein-DNA stoichiometry experiments, the fluorescently labeled and annealed probe (designated *frmRA*Pro) was diluted to 2.5 μm in 10 mm HEPES, pH 7.0, 60 mm NaCl, 240 mm KCl, and 5 mm EDTA and titrated with FrmR or FrmRE64H prepared in 100 mm NaCl, 400 mm KCl, 10 mm HEPES, pH 7.0, and 5 mm EDTA. For *K*_DNA_ determination, *frmRA*Pro was diluted to 10 nm, with addition of 5 mm EDTA or 5 μm ZnCl_2_ as required. FrmR or FrmRE64H was prepared as above with inclusion of 5 mm EDTA or 1.2 m eq of ZnCl_2_ or CuCl (>95% Cu(I)) as appropriate. Changes in anisotropy (Δ*r*_obs_) were measured using a modified Cary Eclipse fluorescence spectrophotometer (Agilent Technologies) fitted with polarizing filters (λ_ex_ = 530 nm, λ_em_ = 570 nm, averaging time = 20 s, replicates = 5, and *T* = 25 °C) as described previously (11). Upon each addition, the cuvette was allowed to equilibrate for 5 min before recording data. Data were fit to the model described in the figure legends and Table 2 footnotes using Dynafit (43). For experiments with Cu(I)- or Zn(II)-FrmR or FrmRE64H, where DNA binding did not saturate, the average fitted Δ*r*_obs_ maximum value from apoprotein experiments was used in the script. The coupling free energy Δ*G*_C_, linking DNA binding to metal binding, was calculated as described previously (11) using the following: Δ*G*_C_ = −*RT*ln*K*_C_, where *r* = 8.314 J K^−1^ mol^−1^ (gas constant), *T* = 298.15 K (temperature at which experiment was conducted), and *K*_C_ = *K*_DNA_^metal-protein^/*K*_DNA_^apoprotein^ (9). Mean Δ*G*_C_ values (and standard deviations) were calculated from the full set of (equally weighted) possible pairwise permutations of *K*_C_.

##### Fractional Occupancy Models

Fractional occupancy of the tightest metal-binding site of a sensor with metal as a function of buffered [metal], was determined using the following: (θ) = [metal]_buffered_/(*K*_metal_ + [metal]_buffered_). *K*_metal_ = *K_D_* (tightest site) of sensor for metal, experimentally determined (*K*_metal_^sensor^) (Table 1) (48). For FrmR (and variants), *K*_metal_ was additionally calculated for the DNA-bound form (*K*_metal_^sensor·DNA^) from the coupling constant (*K*_C_) (Fig. 10*E*). The concentration of apo- and Zn(II)-protein at a given [Zn(II)] was calculated using the number of tetramers per cell (FrmR and variants; Fig. 9*K*), and a cell volume of 1 fl. Fractional DNA occupancies with apo- and Zn(II)-protein over a range of protein concentrations were modeled using Dynafit (43) (1:1 binding of tetramer/DNA; assuming the binding of one tetramer conferred repression) with *K*_DNA_ (from Table 2) and [P*_frmRA_*] as fixed parameters (sample Dynafit script is also shown in the supplemental material). [P*_frmRA_*] was calculated assuming 15 copies cell^−1^ (due to the presence on low copy number reporter plasmid) and a cell volume of 1 fl. The response was set at 1/[P*_frmRA_*]. The fractional occupancy of P*_frmRA_* with apo- and Zn(II)-protein was summed to give fractional occupancy of P*_frmRA_* at any given buffered [Zn(II)].

## Results

### 

#### 

##### CsoR/RcnR-like Repressor FrmR Solely Detects Formaldehyde and Not Metals

**FIGURE 2.**
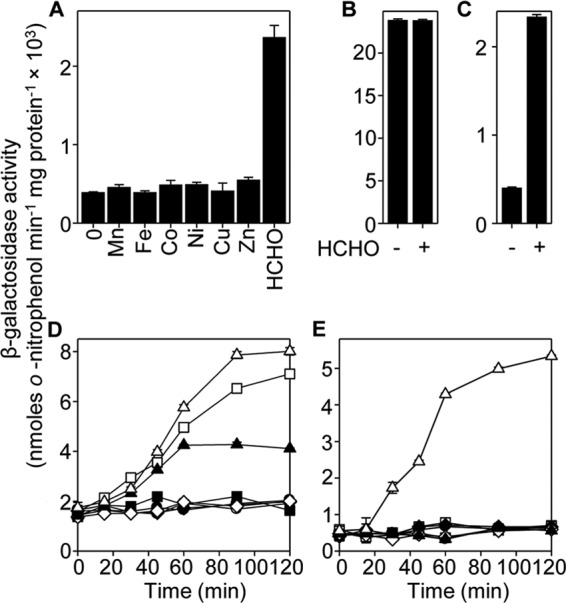
**Single residue change renders *Salmonella* FrmR responsive to cobalt and Zn(II), in addition to formaldehyde.** *A*, β-galactosidase activity in Δ*frmR* containing P*_frmRA_-frmR* fused to *lacZ* grown to mid-exponential phase in M9 minimal medium in the absence or presence of MNIC MnCl_2_, C_6_H_5_FeO_7_, CoCl_2_, NiSO_4_, CuSO_4_, ZnSO_4_, or formaldehyde (HCHO). Expression from P*_frmRA_* (*B*) or P*_frmRA_-frmR* (*C*) following growth to mid-exponential phase in the absence or presence of MNIC formaldehyde is shown. Expression from P*_frmRA_-frmRE64H* (*D*) or P*_frmRA_-frmR* (*E*) as a function of time following exposure of logarithmic cells to MNIC Mn(II) (*open circles*), Fe(III) (*filled squares*), Co(II) (*open squares*), Ni(II) (*filled diamonds*), Cu(II) (*open diamonds*), Zn(II) (*filled triangles*), formaldehyde (*open triangles*), or untreated control (*filled circles*).

Despite similarity between FrmR and metal-sensing transcriptional de-repressors, exposing *Salmonella* cultures to maximum noninhibitory concentrations of MnCl_2_, C_6_H_5_FeO_7_, CoCl_2_, NiSO_4_, CuSO_4_ or ZnSO_4_ does not de-repress expression from P*_frmRA_-frmR* fused to *lacZ* in Δ*frmR* cells (Fig. 2*A*). Exposure of cells to MNIC of formaldehyde does de-repress expression from P*_frmRA_-frmR* (Fig. 2*A*). The formaldehyde response was lost, and basal expression was elevated in cells harboring a similar construct (P*_frmRA_*) devoid of *frmR* (Fig. 2, *B* and *C*). Thus, in common with *E. coli* FrmR (30), the *Salmonella* homologue represses expression from the *frmRA* operator-promoter with repression alleviated by formaldehyde, and here we show that repression by *Salmonella* FrmR is not alleviated by metals.

##### Substitution of FrmR Glu-64 for an RcnR Metal-Ligand Confers Zn(II) and Cobalt Detection in Cells

Replacement of FrmR residue 64 (glutamate) with histidine (a metal-ligand in RcnR; Fig. 1, *A* and *B*) generates a metal-sensing variant of FrmR (Fig. 2*D*). Repression is alleviated by CoCl_2_ and ZnSO_4_ in Δ*frmR* cells containing P*_frmRA_-frmRE64H* (but not P*_frmRA_-frmR*) fused to *lacZ* (Fig. 2, *D* and *E*). MnCl_2_, C_6_H_5_FeO_7_, NiSO_4_, and CuSO_4_ did not affect expression from P*_frmRA_-frmRE64H* (or P*_frmRA_-frmR*), although formaldehyde responsiveness was retained. Notably, metal-responsive family members RcnR and CsoR do respond to nickel and copper (15–17). In summary, a single residue change that mimics the metal-sensing site of RcnR is sufficient to create a detector of cellular Zn(II) and cobalt.

##### FrmRE64H and FrmR Both Bind Co(II), Cu(I), and Zn(II)

**FIGURE 3.**
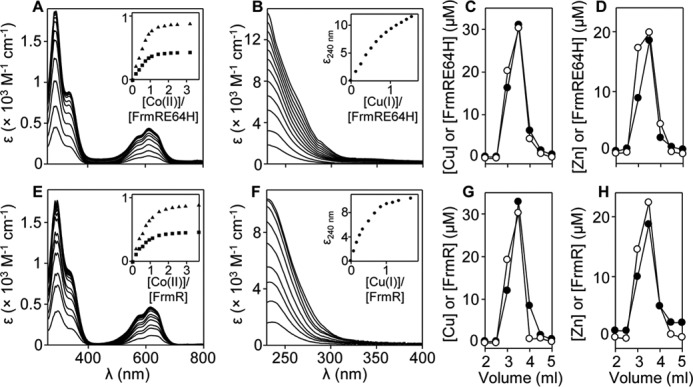
**FrmRE64H and FrmR bind Co(II), Cu(I), and Zn(II).** Apo-subtracted UV-visible difference spectra of FrmRE64H (87.0 μm, monomer) upon titration with CoCl_2_ and binding isotherms (*inset*) at 336 nm (*triangles*) and 614 nm (*squares*) (*A*). FrmRE64H (21.5 μm, monomer) upon titration with CuCl and binding isotherm (*inset*) at 240 nm (*B*). *C*, analysis of fractions (0.5 ml) for protein by Bradford assay (*open circles*) and metal by ICP-MS (*filled circles*) following size exclusion chromatography of FrmRE64H (0.5 ml at 50 μm, monomer) preincubated with 150 μm CuCl. *D*, as *C* except with 38 μm FrmRE64H and 150 μm ZnCl_2_. *E–H*, as described for *A–D* but using FrmR; 83.9 μm (*E*); 21.3 μm (*F*), 50 μm (*G*); and 38 μm (*H*).

It was anticipated that the introduced histidine residue created a metal-binding site in FrmR. However, titration of FrmRE64H or FrmR with Co(II) results in the appearance of spectral features in the region of 330 nm, indicative of S→Co(II) ligand-to-metal charge transfer (LMCT) bands consistent with Co(II) binding to both proteins (Fig. 3, *A* and *E*). For FrmR and FrmRE64H, the intensities of the feature at saturation ∼0.9 × 10^3^ m^−1^ cm^−1^ are consistent with a single thiolate ligand (50). The intensities of a second set of Co(II)-dependent features in the region of 600 nm, indicative of *d-d* transitions (50), suggest tetrahedral coordination geometry. Binding curves are linear up to 1 eq of Co(II), implying *K*_Co(II)_ is too tight to estimate by this method (Fig. 3, *A* and *E*, *insets*). Cu(I)-dependent features similarly indicate tight binding of at least 1 eq of metal, and 1 eq of Cu(I) binds sufficiently tightly to co-migrate with either protein during size exclusion chromatography (Fig. 3, *B, C, F,* and *G*). One equivalent of Zn(II) (which is spectrally silent) also co-migrates with each protein during size exclusion chromatography (Fig. 3, *D* and *H*). Preliminary Ni(II)-binding experiments with FrmRE64H were ambiguous, but because no *in vivo* nickel response had been detected for FrmRE64H, Ni(II) affinities were not pursued.

##### Determination of K_Zn(II)_, K_Co(II)_, and K_Cu(I)_ for FrmRE64H and FrmR

**FIGURE 4.**
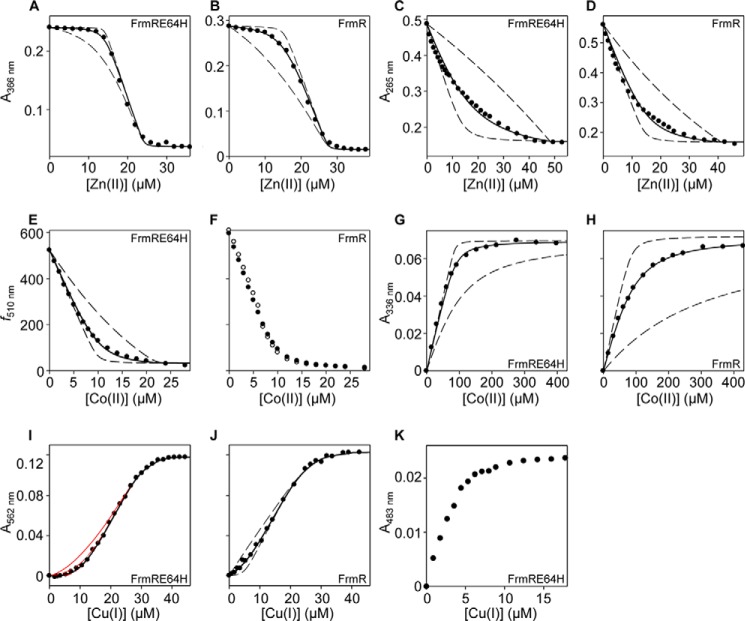
**Zn(II), Co(II), and Cu(I) affinities of FrmRE64H and FrmR.** *A*, representative (*n* = 3) mag fura-2 absorbance upon titration of mag fura-2 (10.1 μm) with ZnCl_2_ in the presence of FrmRE64H (18.8 μm, monomer). *B*, as *A* but with mag fura-2 (12.2 μm) and FrmR (20.4 μm, monomer). *C*, representative (*n* = 3) quin-2 absorbance upon titration of quin-2 (13.4 μm) with ZnCl_2_ in the presence of FrmRE64H (42.7 μm, monomer). *D*, as *C* but with quin-2 (14.1 μm) and FrmR (39.9 μm, monomer). In each case (*A–D*), *solid lines* are fits to a model describing protein competition with mag fura-2 or quin-2 for 0.75 equivalents of Zn(II) per monomer (three sites per tetramer, *K*_Zn1–3_). *Dashed lines* are simulated curves with *K*_Zn1–3_ 10-fold tighter and 10-fold weaker. *E,* representative (*n* = 3) fura-2 fluorescence emission (λ_ex_ = 360 nm) upon titration of fura-2 (9.8 μm) with CoCl_2_ in the presence (*filled circles*) of FrmRE64H (49.3 μm, monomer). *Solid line* is a fit to a model describing protein competition for 0.25 eq of Co(II) per monomer (one site per tetramer, *K*_Co1_). *Dashed lines* are simulated curves describing *K*_Co1_ 10-fold tighter and 10-fold weaker. *F,* fluorescence emission of fura-2 (10.3 μm) upon titration with Co(II) as described in *E* in the absence (*open circles*) or presence (*filled circles*) of FrmR (41.9 μm, monomer). *G*, representative (*n* = 3) Co(II)-dependent absorbance at 336 nm of FrmRE64H (87.0 μm, monomer) upon titration with CoCl_2_ in the presence of 50 mm BisTris. *H*, as *G* but with FrmR (83.9 μm, monomer). *Solid lines* (for *G* and *H*), are fits to a model describing protein competition for 1 m eq of Co(II) per monomer (four sites per tetramer, *K*_Co1–4_). *Dashed lines* are simulated curves describing *K*_Co1–4_ 10-fold tighter and 10-fold weaker. *I*, representative (*n* = 3) BCA absorbance upon titration of BCA (40 μm) with CuCl in the presence of FrmRE64H (11 μm, monomer). *J*, as *I* but with FrmR (10 μm, monomer). *Solid lines* (for *I* and *J*) are fits to a model describing protein competition with BCA for 2 eq of Cu(I) per monomer (eight sites per tetramer). *Dashed lines* are simulated curves with *K*_Cu1–2_ 10-fold tighter and 10-fold weaker. *Solid red line* (*I* only) is a simulated curve describing *K*_Cu1–2_ 100-fold weaker. *K*, representative (*n* = 4) BCS absorbance upon titration of BCS (10 μm) with CuCl in the presence of FrmRE64H (29.7 μm, monomer).

As the FrmRE64H variant, but not FrmR, responds to Zn(II) and cobalt in cells, it was anticipated that this substitution had succeeded in tightening the affinity for these metals. The chromophores mag fura-2 and quin-2 form 1:1 complexes with Zn(II) and undergo concomitant changes in absorbance upon metal binding, which can be used to monitor competition with proteins and hence to estimate protein *K*_Zn(II)_ (11, 13, 44–48, 51). Titration of 10.1 μm or 12.2 μm mag fura-2 with Zn(II) in the presence of FrmRE64H (18.8 μm, monomer) or FrmR (20.4 μm, monomer), respectively, gave negligible change in absorbance up to 0.5–0.75 eq of Zn(II) per protein monomer, implying competition with the chromophore for metal (Fig. 4, *A* and *B*). At these protein concentrations, CsoR/RcnR family members exist as tetramers with four metal-binding sites per tetramer, and with some evidence of negative cooperativity between sites (12, 17, 18, 52). A 1:1 stoichiometry equating to four Zn(II) per tetramer was observed for both FrmRE64H and FrmR (Fig. 3, *D* and *H*), but the fourth sites are too weak to compete with mag fura-2 (hence competition is complete after addition of ∼24.2 and ∼27.5 μm Zn(II) (Fig. 4, *A* and *B*)). Data were fit to models describing tight binding of 3 m eq of Zn(II)/tetramer, with *dashed lines* representing simulated curves describing *K*_Zn1–3_ 10-fold tighter or 10-fold weaker than the calculated affinity (Fig. 4, *A* and *B*). For both proteins, this suggests *K*_Zn1–3_ at or approaching the tighter limit of the assay using mag fura-2 (*K*_Zn(II)_^mag fura-2^ = 2.0 × 10^−8^ m). Competitions were therefore conducted with 13.4 or 14.1 μm quin-2 (*K*_Zn(II)_^quin-2^ = 3.7 × 10^−12^ m) and FrmRE64H (42.7 μm, monomer) or FrmR (39.9 μm, monomer), respectively (Fig. 4, *C* and *D*). Again, data were fit to models describing binding of 3 m eq of Zn(II)/tetramer (as expected, the fourth sites did not show competition with quin-2) with *dashed lines* in Fig. 4, *C* and *D,* describing simulated curves for *K*_Zn1–3_ 10-fold tighter or 10-fold weaker than the calculated affinity of the proteins. Mean values of *K*_Zn1–3_ 2.33 (±0.3) × 10^−11^ m and 1.7 (±0.7) × 10^−10^ m for FrmRE64H and FrmR, respectively, are thus within the range of this assay (Fig. 4, *C* and *D*, and Table 1).

**TABLE 1:** **Metal affinities of FrmR, FrmRE64H, Zur, ZntR, RcnR, and CueR** The following conditions were used: 10 mm HEPES, pH 7.0, 100 mm NaCl, 400 mm KCl for FrmR, FrmRE64H, Zur, ZntR, and CueR; 10 mm HEPES, pH 7.0, 5% glycerol, 100 mm NaCl, 400 mm KCl for RcnR.

Sensor	Metal	*K*_metal_ (m)
FrmR	Co(II)	*K*_1–4_*^a^* = 7.59 ± 0.4 × 10^−6^
	Zn(II)	*K*_1–3_*^b^* = 1.7 ± 0.7 × 10^−10^
	Cu(I)	*K*_1–2_*^c^* = 4.9 ± 1.6 × 10^−15^; *K*_3–4_*^c^* = 1.72 ± 0.7 × 10^−12^; *K*_5–8_*^c^* ≥8 × 10^−11^
FrmRE64H	Co(II)	*K*_1_*^d^* = 2.56 ± 0.4 × 10^−7^; *K*_1–4_*^a^* <10^−6^
	Zn(II)	*K*_1–3_*^b^* = 2.33 ± 0.3 × 10^−11^
	Cu(I)	*K*_1–2_*^e,f^* ∼5 × 10^−16^; *K*_3–4_*^e^* = 7.29 ± 1.29 × 10^−15^; *K*_5–6_*^e^* = 5.6 ± 2.0 × 10^−12^; *K*_7–8_*^e^* ≥4 × 10^−10^
Zur	Zn(II)	*K*_1–2_*^g^* = 6.36 ± 0.41 × 10^−13^; *K*_3_*^g^* = 8.04 ± 2.92 × 10^−11^; *K*_4_*^h^* ≥5 × 10^−7^
ZntR	Zn(II)	*K*_1_*^i^* = 3.2 ± 0.73 × 10^−12^; *K*_2_*^i^* = 2.68 ± 0.73 × 10^−11^
RcnR	Co(II)	*K*_1–2_*^j^* = 5.06 ± 0.86 × 10^−10^; 3 × 10^−5^ ≥*K*_3_*^j,k^* ≥10^−7^
CueR	Cu(I)	*K*_1_*^l^* = 3.25 ± 0.66 × 10^−19^

*^a^* Data were fit to a model describing Co(II) binding with equal affinity to four sites (*K*_Co1–4_) on an FrmR or FrmRE64H tetramer, determined by competition with BisTris (*n* = 3). A weaker limit is defined for FrmRE64H.

*^b^* Data were fit to a model describing Zn(II) binding with equal affinity to the first three sites (*K*_Zn1–3_) on an FrmR or FrmRE64H tetramer, determined by competition with quin-2 (*n* = 3).

*^c^* Data were fit to a model describing Cu(I) binding with equal affinity to the first two sites (*K*_Cu1–2_), with equal affinity to sites 3 and 4 (*K*_Cu3–4_), and with equal affinity to sites 5–8 (*K*_Cu5–8_) on an FrmR tetramer (with *K*_Cu1–2_ < *K*_Cu3–4_ < *K*_Cu5–8_), determined by competition with BCA (*n* = 4). A tighter limit is defined for FrmR *K*_Cu5–8_.

*^d^* Fit to a model describing Co(II) binding to the first site (*K*_Co1_) on an FrmRE64H tetramer, determined by competition with fura-2 (*n* = 3).

*^e^* Data were fit to a model describing Cu(I) binding with equal affinity to the first two sites (*K*_Cu1–2_), with equal affinity to sites 3 and 4 (*K*_Cu3–4_), with equal affinity to sites 5 and 6 (*K*_Cu5–6_), and with equal affinity to sites 7 and 8 (*K*_Cu7–8_) on an FrmRE64H tetramer (with *K*_Cu1–2_ < *K*_Cu3–4_ < *K*_Cu5–6_ < *K*_Cu7–8_), determined by competition with BCA (*n* = 4). A tighter limit is defined for FrmRE64H *K*_Cu7–8_.

*^f^* Approximation reflects the fact that sites 1 and 2 on an FrmRE64H tetramer outcompete BCA for Cu(I) but fail to compete with BCS (although the formation of a ternary complex cannot be ruled out).

*^g^* Data were fit to a model describing Zn(II) binding to three sites (*K*_Zn1–2_ and *K*_Zn3_) on a Zur dimer (with the structural site already filled) with equal affinity to the first two sites, (*K*_Zn1–2_) and *K*_Zn1–2_ < *K*_Zn3_, determined by competition with quin-2 (*n* = 3).

*^h^* Data were fit to a model describing Zn(II) binding to the fourth site (*K*_Zn4_) on a Zur dimer (with the structural site already filled), determined by competition with mag fura-2 (*n* = 3). Only a tighter limit can be determined.

*^i^* Data were fit to a model describing Zn(II) binding to two sites (*K*_Zn1_ and *K*_Zn2_) on a ZntR dimer (*K*_Zn1_ < *K*_Zn2_), determined by competition with quin-2 (*n* = 3).

*^j^* Data were fit to a model describing Co(II) binding to three sites (*K*_Co1–2_ and *K*_Co3_) on an RcnR tetramer with equal affinity to the first two sites (*K*_Co1–2_) and *K*_Co1–2_ < *K*_Co3_, determined by competition with fura-2 (*n* = 3).

*^k^* Range represents the fact that RcnR exhibits linear absorbance features upon titration with Co(II) to 1 m eq per monomer, but site 3 does not sufficiently complete with fura-2 for Co(II).

*^l^* Data were determined by competition with BCS (*n* = 6) and describing binding of Cu(I) to the first site (*K*_Cu1_) on a CueR dimer.

Co(II) affinities of FrmRE64H (49.3 μm, monomer) and FrmR (41.9 μm, monomer) were first analyzed by competition with 10.3 and 9.8 μm fura-2, respectively (*K*_Co(II)_^fura-2^ = 8.64 × 10^−9^ m) (Fig. 4, *E* and *F*) (6, 47). Only FrmRE64H showed competition with fura-2 (Fig. 4*E*). The data were fit to a model describing binding of one Co(II) ion per FrmRE64H tetramer, which significantly departs from simulated curves describing *K*_Co1_ 10-fold tighter (Fig. 4*E*), with *K*_Co1_ from triplicate assays = 2.56 (±0.4) × 10^−7^ m (Table 1). In contrast, both FrmRE64H (87.0 μm, monomer) and FrmR (83.9 μm, monomer) showed competition with a large excess (50 mm) of BisTris (*K*_Co(II)_^BisTris^ = 2.26 × 10^−2^ m) (Fig. 4, *G* and *H*) (48, 53). The data were fit to a model describing binding of four Co(II) ions, with equal affinity, per tetramer, which significantly departs from simulated curves describing *K*_Co1–4_ as 10-fold weaker. For FrmR, the curves also depart from simulated curves describing *K*_Co1–4_ as 10-fold tighter. *K*_Co1–4_ from triplicate assays = 7.59 (±0.4) × 10^−6^ m for FrmR, whereas only a weaker limit (<10^−6^ m) for FrmRE64H *K*_Co1–4_ could be determined (Table 1).

Cuprous affinities of both proteins were determined using BCA (β_2_ = 10^17.2^ m^−2^ (48)) revealing competition in each case for 2 m eq of Cu(I) per monomer, but with greater competition and hence tighter affinity for FrmRE64H than FrmR (Table 1 and Fig. 4, *I* and *J*). The data were fit to models describing binding of eight Cu(I) ions per tetramer (see Table 1 footnotes for details), which for FrmR depart from simulated curves describing binding of the tightest two Cu(I) ions (*K*_Cu1–2_) 10-fold tighter and 10-fold weaker than the fitted value (Fig. 4*I*), giving FrmR *K*_Cu1–2_ = 4.9 (±1.6) × 10^−15^ m (Table 1). In contrast, *K*_Cu1–2_ for FrmRE64H is too tight to measure by this assay. However, FrmRE64H does not significantly compete with 10 μm BCS (β_2_ = 10^19.8^ m^−2^) (48), with saturation of the BCS_2_Cu(I) complex observed at ∼5 μm CuCl (Fig. 4*K*). These data imply that FrmRE64H *K*_Cu1–2_ can only marginally depart from the value estimated using BCA (*K*_Cu1–2_ ∼5 × 10^−16^ m) (Table 1). It is noted that the final absorbance for the BCS_2_Cu(I) complex in the presence of protein was lower than predicted from its known extinction coefficient; hence, the possibility of a ternary complex cannot be ruled out. In summary, the two metals that FrmRE64H now detects, Co(II) and Zn(II), bind approximately an order of magnitude more tightly than to FrmR (Table 1).

##### Cognate K_metal_ of Salmonella Zn(II), Cobalt, and Cu(I) Sensors ZntR, Zur, RcnR, and CueR

**FIGURE 5.**
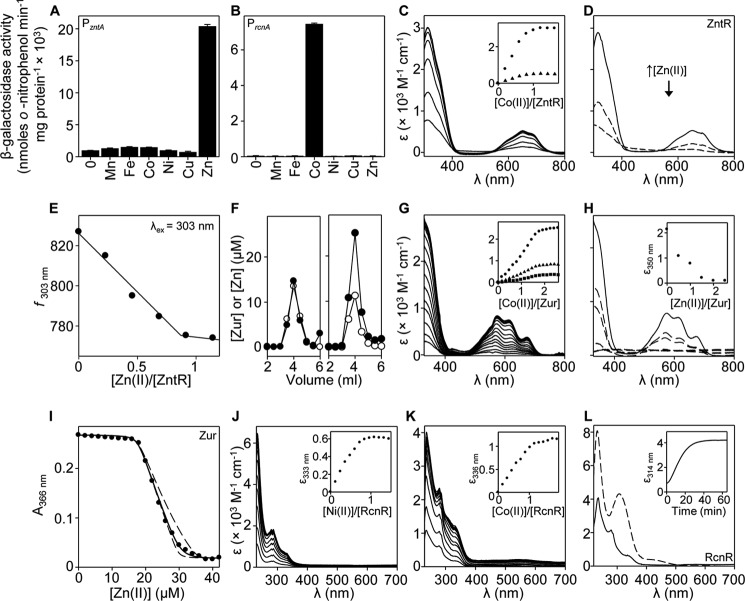
**Characterization of *Salmonella* ZntR, Zur, and RcnR.** β-Galactosidase activity in wild type *Salmonella* (defined earlier) containing P*_zntA_* (*A*) or *rcnR-*P*_rcnA_* (*B*) fused to *lacZ* following growth to mid-exponential phase in the absence or presence of MNIC MnCl_2_, C_6_H_5_FeO_7_, CoCl_2_, NiSO_4_, CuSO_4_, or ZnSO_4_. *C*, apo-subtracted UV-visible difference spectra of ZntR (24.9 μm, monomer) upon titration with CoCl_2_. *Inset,* binding isotherms at 314 nm (*circles*) and 650 nm (*triangles*). *D*, apo-subtracted UV-visible difference spectra of Co(II)-ZntR (24.0 μm, monomer; equilibrated with 1 m eq CoCl_2_) (*solid line*), and following addition of 0.5 and 1 m eq of ZnCl_2_ (*dashed lines*). *E*, fluorescence emission of ZntR (13.1 μm, monomer) following titration with ZnCl_2_. *F*, analysis of fractions (0.5 ml) for protein by Bradford assay (*open circles*) and zinc by ICP-MS (*filled circles*) following size exclusion chromatography of Zur (0.5 ml at 20 μm, monomer) preincubated with 1 mm EDTA (*left panel*) or 120 μm ZnCl_2_ (*right panel*). *G*, apo-subtracted UV-visible difference spectra of Zur (24.8 μm, monomer) upon titration with CoCl_2_. *Inset,* binding isotherms at 350 nm (*circles*), 576 nm (*triangles*), and 670 nm (*squares*). *H*, apo-subtracted UV-visible difference spectra of Zur (27.7 μm, monomer; equilibrated with 2 m eq of CoCl_2_) (*solid line*) and following titration with ZnCl_2_ (*dashed lines*). *Inset,* quenching of feature at 350 nm. *I*, representative (*n* = 3) mag fura-2 absorbance upon titration of mag fura-2 (12.1 μm) with ZnCl_2_ in the presence of Zur (11.7 μm, monomer). *Solid line* describes competition from Zur for 2 eq of Zn(II) per monomer (four exchangeable sites per dimer, with three independent binding events: *K*_Zn1–2,_ *K*_Zn3_, and *K*_Zn4_). *Dashed lines* are simulated curves with *K*_Zn4_ 10-fold tighter and 10-fold weaker than fitted *K*_Zn4_ (*K*_Zn1–2_ and *K*_Zn3_ fixed to fitted values). *J*, apo-subtracted UV-visible difference spectra of RcnR (30.6 μm, monomer) upon titration with NiCl_2_. *Inset,* binding isotherm at 333 nm. *K*, as *J* except with RcnR (27.3 μm, monomer) and CoCl_2_. *Inset,* binding isotherm at 336 nm. *L*, apo-subtracted absorbance of RcnR (31.4 μm, monomer) after addition of 34.5 μm CoCl_2_ and incubation at room temperature under anaerobic conditions in a gas-tight cuvette for 10 min (*solid line*) or 65 h (*dashed line*). *Inset,* time course at 314 nm.

**FIGURE 6.**
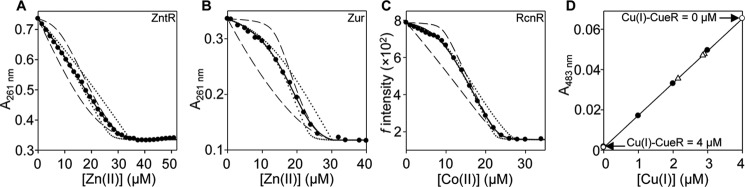
**Cognate metal affinities of *Salmonella* ZntR, Zur, RcnR, and CueR.** *A*, representative (*n* = 3) quin-2 absorbance upon titration of quin-2 (18.6 μm) and ZntR (16.0 μm, monomer) with ZnCl_2_. *Solid line* describes competition from ZntR for 1 m eq of Zn(II) per monomer (two independent sites per dimer; *K*_Zn1_ and *K*_Zn2_). *Dashed lines* describe *K*_Zn1_ 10-fold tighter and 10-fold weaker than fitted *K*_Zn1_ (*K*_Zn2_ fixed to fitted *K*_Zn2_). *Dotted lines* describe *K*_Zn2_ 10-fold tighter and 10-fold weaker than fitted *K*_Zn2_ (*K*_Zn1_ fixed to fitted *K*_Zn1_). *B,* representative (*n* = 3) quin-2 absorbance upon titration of quin-2 (9.6 μm) and Zur (13.7 μm, monomer) with ZnCl_2_. *Solid line* describes competition from Zur for 1.5 m eq of Zn(II) per monomer (three sites per dimer with two independent binding events: *K*_Zn1–2_, and *K*_Zn3_). *Dashed lines* describe *K*_Zn1–2_ 10-fold tighter and 10-fold weaker than fitted *K*_Zn1–2_ (*K*_Zn3_ fixed to fitted *K*_Zn3_). *Dotted lines* describe *K*_Zn3_ 10-fold tighter and 10-fold weaker than the fitted *K*_Zn3_ (*K*_Zn1–2_ fixed to fitted *K*_Zn1–2_). *C*, representative (*n* = 3) fura-2 fluorescence emission upon titration of fura-2 (13.2 μm) and RcnR (18.4 μm, monomer) with CoCl_2_. *Solid line* describes competition from RcnR for 0.75 m eq of Co(II) per monomer (three sites per tetramer, with two independent binding events: *K*_Co1–2_ and *K*_Co3_). *Dashed lines* describe *K*_Co1–2_ 10-fold tighter and 10-fold weaker than the fitted *K*_Co1–2_ (*K*_Co3_ fixed to fitted *K*_Co3_). *Dotted lines* describe *K*_Co3_ 10-fold tighter and 10-fold weaker than the fitted *K*_Co3_(*K*_Co1–2_ fixed to fitted *K*_Co1–2_). *D*, absorbance at 483 nm of BCS (750 μm) titrated with CuCl (*filled circles*) or BCS (750 μm) pre-equilibrated with 4 μm Cu(I) and incubated (60 min) with CueR (12.3 μm, monomer) (*n* = 3, *open triangles*). Absorbance values depicting complete, or no competition from CueR for Cu(I) are shown (*open circles*).

If metal sensing is dictated by relative affinity within the set of *Salmonella* metal sensors, the affinity of FrmRE64H for Zn(II) and Co(II) would need to become comparable with cellular sensors for these metals. Conversely, Cu(I) affinity would need to remain weaker than Cu(I)-sensing CueR making Cu(I) still undetectable (40, 41, 54). The *Salmonella* sensors for Zn(II) and Co(II) are confirmed here as ZntR, Zur, and RcnR (Fig. 5) (55, 56). Expression is induced from P*_zntA_* and P*_rcnA_* in wild type cells exposed to MNIC ZnSO_4_ and CoCl_2_, respectively (Fig. 5, *A* and *B*). Notably minimal media for this strain (SL1344) require histidine that may influence Ni(II) availability. Titration of ZntR with Co(II), as a spectral probe for Zn(II)-binding sites, generated features diagnostic for LMCTs and *d-d* transitions consistent with ∼3 thiolate-Co(II) bonds per ZntR monomer and tetrahedral coordination geometry (Fig. 5*C*) (50). These features saturate at ∼1 eq of Co(II) and are bleached by addition of ∼1 eq of Zn(II) (Fig. 5*D*). Zn(II) (∼1 eq) also quenched ZntR auto-fluorescence (Fig. 5*E*). *Salmonella* ZntR is expected to be a dimer based on similarity to the *E. coli* homologue (49), implying a stoichiometry of two Zn(II) ions per dimer. Titrations of 18.6 μm quin-2 and ZntR (16.0 μm, monomer) with Zn(II) were fit to models describing detectable binding of two distinguishable Zn(II) ions per dimer (*K*_Zn1_ and *K*_Zn2_); the estimated mean values are shown in Table 1 (Fig. 6*A*). The optimized curves depart from simulated curves describing *K*_Zn1_ or *K*_Zn2_ 10-fold tighter or 10-fold weaker than their fitted values, although *K*_Zn2_ does approach the simulated curve describing *K*_Zn2_ as 10-fold weaker (Fig. 6*A*). It remains possible that a higher Zn(II) stoichiometry may be achieved for *Salmonella* ZntR under some conditions (as observed for *E. coli* ZntR (49)). Importantly, we show here that ZntR binds only two Zn(II) ions per dimer with sufficient affinity to compete with quin-2.

Zur from *Salmonella*, and in common with other bacteria (57–60), contains a structural Zn(II) ion that remains associated with the protein (20 μm, monomer) in the presence of excess (1 mm) EDTA (Fig. 5*F*). In the absence of EDTA, at least one further equivalent of Zn(II) binds sufficiently tightly to co-migrate with the protein during size exclusion chromatography (Fig. 5*F*). Titration of apo-Zur (Zn(II)-saturated at the structural site) with Co(II) generated features diagnostic for LMCTs and *d-d* transitions consistent with two to three coordinating thiol groups, which saturate between 1.5 to 2 eq of Co(II) per monomer (Fig. 5*G*) (50). These features are bleached with addition of 1.5 to 2 eq of Zn(II) (Fig. 5*H*). Zur family members exist as dimers (57–59), and here data show there are at least three exchangeable sites per dimer that are accessible to both Co(II) and Zn(II). A total of 35.5 μm Zn(II) is required to fully saturate Zur (11.7 μm, monomer) and mag fura-2 (12.1 μm), consistent with two monomer equivalents ((2 × 11.7 μm) + 12.1 μm = 35.5 μm) of exchangeable Zn(II) binding to Zur (∴ four sites per dimer) with sufficient affinity to show some competition with mag fura-2. Of these, an estimated three sites per dimer completely withhold Zn(II) from mag fura-2 (Fig. 5*I*). The data in Fig. 5*I* were fit to a model describing four exchangeable sites per Zur dimer with *dashed lines* representing simulated curves describing *K*_Zn4_ 10-fold tighter and 10-fold weaker than the fitted *K*_Zn4_ value, and a tighter limit for *K*_Zn4_ was estimated from replicate titrations (Table 1). To estimate *K*_Zn1–2_ and *K*_Zn3_, quin-2 (9.6 μm) and Zur (13.7 μm, monomer) were titrated with Zn(II) and fit to models describing competition from 1.5 eq of Zn(II) per monomer (exchangeable sites 1–3 per dimer, but not site 4) with mean values for *K*_Zn1–2_ and *K*_Zn3_ shown in Table 1 (Fig. 6*B*). The optimized curve departs from simulated curves describing *K*_Zn1–2_ as 10-fold tighter or 10-fold weaker than the fitted value. *K*_Zn3_ departs from a simulated curve describing *K*_Zn3_ as 10-fold tighter, but it approaches a simulated curve describing *K*_Zn3_ as 10-fold weaker (Fig. 6*B*).

Titration of RcnR with Ni(II) or Co(II) generated spectral features that saturated at 1 eq of metal (Fig. 5, *J* and *K*). Ni(II)-RcnR demonstrated features <300 nm and weak *d-d* transitions consistent with a six coordinate octahedral Ni(II)-binding site, as seen for *E. coli* RcnR (17). An additional Co(II)-dependent feature at 314 nm appeared with time (Fig. 5*L*). Co(II)-dependent fluorescence quenching of fura-2 (13.2 μm) in the presence of RcnR (18.4 μm, monomer) was fit to a model describing competition from three sites per RcnR tetramer with two sites (*K*_Co1–2_) tighter than the third (*K*_Co3_) (Fig. 6*C*). The optimized curve departs from simulated curves describing *K*_Co1–2_ as 10-fold tighter or 10-fold weaker and *K*_Co3_ as 10-fold tighter than the respective fitted values. Mean values (generated from multiple titrations) for *K*_Co1–2_ and a range for *K*_Co3_ are shown in Table 1.

*Salmonella* CueR out-competes a 10-fold molar excess of BCS (41), and here a 100-fold and then a 75-fold excess of BCS (the latter in Fig. 6*D*) were used to estimate *K*_Cu1_ (Table 1). In summary, the tightest exchangeable sites of the endogenous metal sensors are tighter for their cognate metals than either FrmR or FrmRE64H, in every case (Table 1). However, the difference in *K*_Zn(II)_ between FrmRE64H and cognate Zn(II) sensors is the smallest.

##### Cognate Metal Sensors Out-compete FrmR for Metal

**FIGURE 7.**
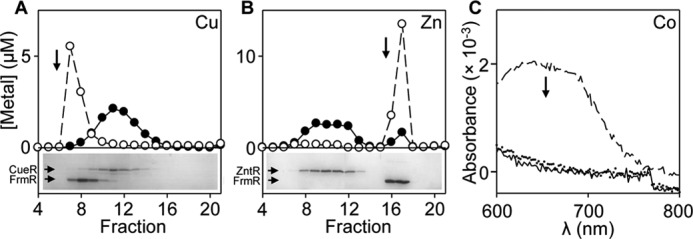
***Salmonella* metal sensors compete with FrmR for their cognate metals.** *A,* heparin affinity chromatography of FrmR (40 μm, monomer) equilibrated with 10 μm CuCl (*open circles*) or with addition of 20 μm CueR, monomer (*filled circles*). *B,* as *A* except with ZnCl_2_ (10 μm) and addition of ZntR instead of CueR. ZntR does not bind the column, and FrmR elutes in later fractions (relative to *A*) at this ionic strength. In each case, fractions (1 ml) were assayed for metal by ICP-MS and protein by SDS-PAGE (shown for the competition experiments). *C*, apo-subtracted difference spectra following addition of 9.9 μm CoCl_2_ to 41.5 μm FrmR monomer (*dashed line*), 42.2 μm RcnR monomer (*dotted line*), or FrmR followed by addition of RcnR (*solid line*).

To confirm, or otherwise, that FrmR *K*_metal_ is weaker than CueR *K*_Cu(I)_, ZntR *K*_Zn(II)_, and RcnR *K*_Co(II)_, pairwise competitions were conducted for the tightest metal-binding site in which metallated FrmR was incubated with apo-forms of the respective sensors. Cu(I)-FrmR co-migrates with copper following heparin affinity chromatography (Fig. 7*A*). However, after mixing Cu(I)-FrmR with apo-CueR (which can be differentially resolved), copper migrates with CueR (Fig. 7*A*). Likewise after mixing Zn(II)-FrmR with apo-ZntR, Zn(II) predominantly migrates (using different fractionation buffers to those in Fig. 7*A*) with ZntR (> 90% of control) (Fig. 7*B*). Diagnostic spectral features (*d-d* transitions) that discern Co(II)-FrmR, with tetrahedral binding geometry, from Co(II)-RcnR, with octahedral binding geometry, are lost upon addition of apo-RcnR to Co(II)-FrmR (Fig. 7*C*). Thus, in every case the cognate sensor out-competes FrmR confirming that FrmR *K*_metal_ is weaker. Relative (to the cognate sensors) metal affinity could account for why wild type FrmR does not respond to metals within cells.

**FIGURE 8.**
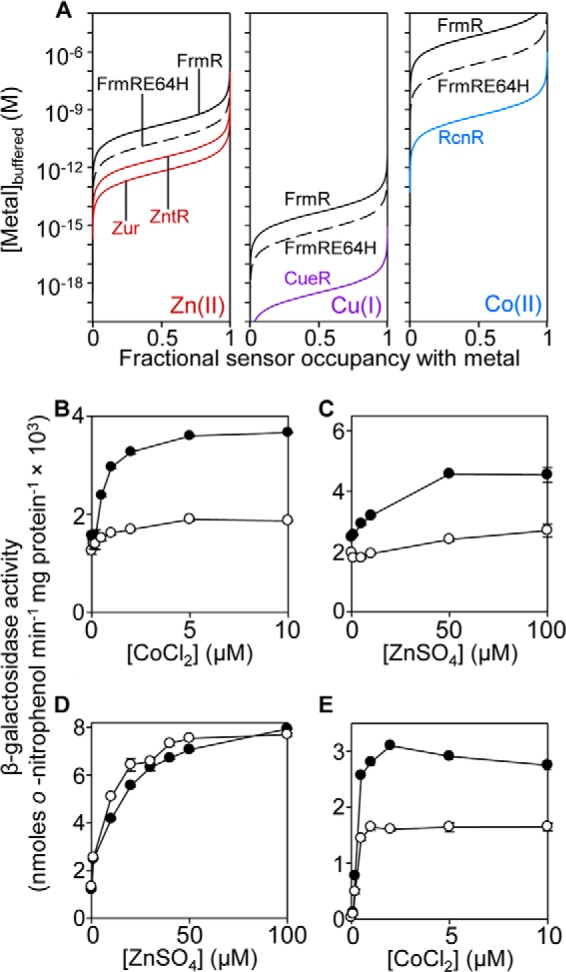
**Comparative metal affinities and the contribution of glutathione to metal sensing.** *A,* fractional occupancy of FrmR and FrmRE64H with Zn(II), Cu(I), and Co(II) as a function of (buffered) metal concentration compared with cognate metal sensors from *Salmonella*: Zur and ZntRwith Zn(II), CueR with Cu(I), and RcnR with Co(II). Fractional occupancy (θ) = [metal]_buffered_/(*K*_metal_ + [metal]_buffered_) using *K*_metal_ in Table 1. *B*, β-galactosidase activity in Δ*frmR* (*filled circles*) or Δ*frmR/*Δ*gshA* (*open circles*) containing P*_frmRA_-frmRE64H* following exposure (2 h) of logarithmic cells to CoCl_2_. *C*, as *B* but with ZnSO_4_ instead of CoCl_2_. *D*, β-galactosidase activity in *Salmonella* (wild type, defined earlier) (*filled circles*) or Δ*gshA* (*open circles*) containing P*_zntA_* grown as described in *C. E,* β-galactosidase activity in wild type (*filled circles*) or Δ*gshA* (*open circles*) containing *rcnR*-P*_rcnR_* following growth in conditions described in *B*.

Fig. 8*A* compares the calculated fractional occupancies of the tightest exchangeable sites (from *K*_metal_ in Table 1) of FrmR and FrmRE64H for Zn(II), Cu(I), and Co(II) with the respective cognate *Salmonella* sensors, as a function of metal concentration. To detect Cu(I), FrmRE64H would require intracellularly buffered Cu(I) concentrations to rise ∼3 orders of magnitude higher than necessary for detection by CueR, which could explain why FrmRE64H remains unresponsive to Cu(I). In contrast, partial Zn(II) occupancy of FrmRE64H will occur at Zn(II) concentrations below those required to saturate ZntR (Fig. 8*A*). Thus, theoretically, a 10-fold increase in *K*_Zn(II)_ of FrmRE64H relative to FrmR may be sufficient to enable some Zn(II) detection within the cell.

##### Glutathione Enhances Metal Detection by FrmRE64H and RcnR

In addition to responding to Zn(II), the FrmRE64H variant also responds to cellular cobalt (Fig. 2*D*), yet *K*_Co(II)_ for FrmRE64H is ∼500-fold weaker than the endogenous cobalt sensor RcnR (Fig. 8*A* and Table 1). An ∼10-fold increase in *K*_Co(II)_ alone cannot readily explain why this variant of FrmR has become responsive to cobalt. Recent studies of the complement of metal sensors from a cyanobacterium concluded that the detection of Zn(II) and nickel matched predictions based upon equilibrium thermodynamics, but this was untrue for cobalt (6, 11, 18). In that system, a substantial kinetic component was invoked for the preferential distribution of cobalt to the cobalt sensor and away from sensors for other metals (6).

The possibility that glutathione is required for the detection of cobalt (and Zn(II)) by FrmRE64H was investigated in Δ*frmR/*Δ*gshA* cells containing P*_frmRA_-frmRE64H* fused to *lacZ* (Fig. 8, *B* and *C*). Cells lacking glutathione showed a negligible response to either metal. Previous studies of Zn(II) sensors have found that the low molecular weight thiol, bacillithiol, competes for metal thus reducing responses (61). ZntR-mediated expression in response to Zn(II) from the *zntA* promoter shows negligible difference in Δ*gshA* cells compared with wild type (Fig. 8*D*). However, in common with regulation by FrmRE64H, the response of RcnR to cobalt was also reduced, but not lost, in cells missing glutathione (Fig. 8*E*). Thus, glutathione aids the detection of cobalt by two different sensors but has varied effects on Zn(II) sensing.

##### Basal Repression by FrmRE64H Is Less than by FrmR

**FIGURE 9.**
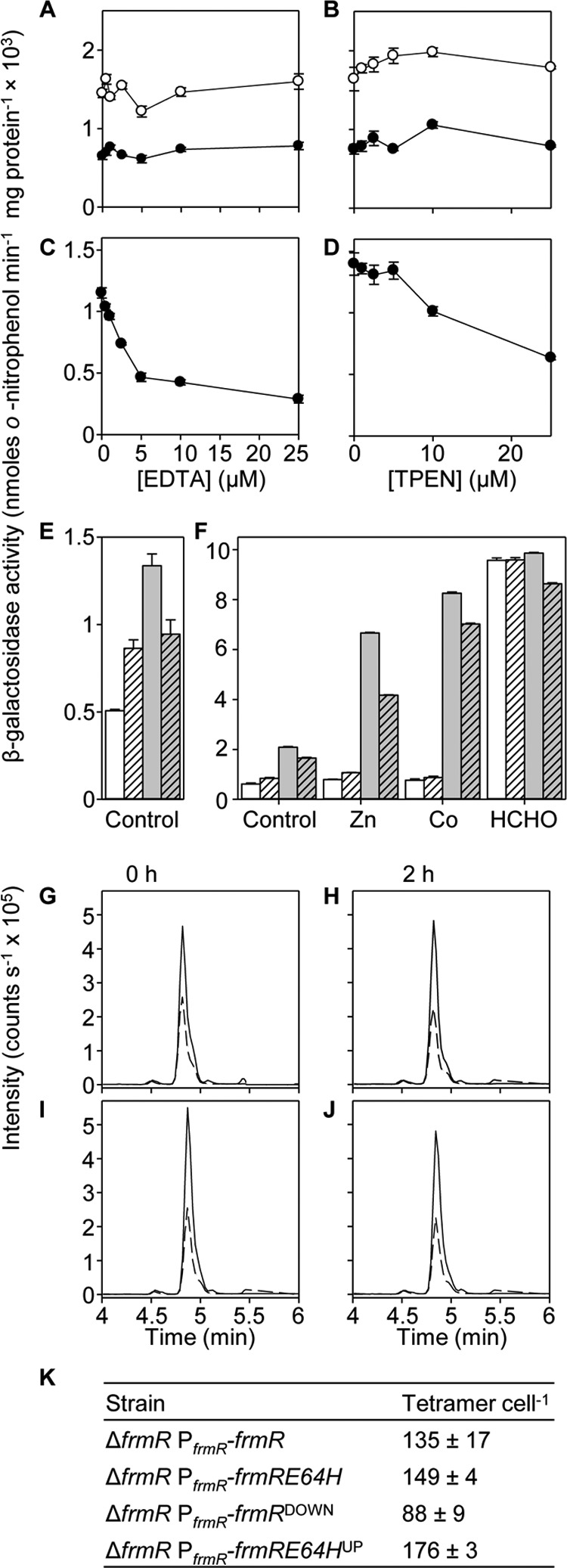
**Basal expression from P*_frmRA_-frmRE64H* is higher than P*_frmRA_-frmR*.** β-Galactosidase activity in Δ*frmR* containing P*_frmRA_-frmR* (*filled circles*) or P*_frmRA_-frmRE64H* (*open circles*) following growth to early exponential phase in the presence of EDTA (*A*) or TPEN (*B*). *C*, expression from P*_zntA_* in wild type *Salmonella*, grown as described in *A*, or *D*, as described in *B. E*, expression in Δ*frmR* containing P*_frmRA_-frmR* (*white bars*), P*_frmRA_-frmR*^DOWN^ (*dashed white bars*), P*_frmRA_-frmRE64H* (*gray bars*), or P*_frmRA_-frmRE64H*^UP^ (*dashed gray bars*) following growth to early exponential phase, and *F*, following exposure (2 h) to Zn(II), Co(II) or formaldehyde, or untreated control. *G–J*, multiple reaction monitoring, quantitative MS of cell extracts. Representative (*n* = 3) extracted LC-MS chromatograms of ion transitions detected in Δ*frmR* containing P*_frmRA_-frmR* (*G*), P*_frmRA_-frmR*^DOWN^ (*H*), P*_frmRA_-frmRE64H* (*I*), or P*_frmRA_-frmRE64H*^UP^ (*J*). Transitions 451.24/716.4 and 456.24/726.4 are for analyte GQVEALER (*solid lines*) or labeled GQVEALER[^13^C_6_,^15^N_4_] ([^13^C_6_,^15^N_4_]arginine residue) (*dashed lines*). *K*, abundance of FrmR and variants using quantitative data obtained in *G–J*.

The tightening of *K*_Zn(II)_ (and *K*_Co(II)_) is modest suggesting that additional factors might contribute to the gain-of-metal detection by FrmRE64H (Figs. 2*D* and 8*A* and Table 1). It was noted that basal expression from the *frmRA* promoter is greater in cells containing FrmRE64H compared with wild type FrmR (Fig. 2, *D* and *E*). Expression remains elevated in cultures treated with EDTA or the Zn(II) chelator TPEN, implying that this is not a response to basal levels of intracellular metal (Fig. 9, *A* and *B*). As a control, ZntR-mediated β-galactosidase expression from the *zntA* promoter does decline upon equivalent treatment with EDTA or TPEN (Fig. 9, *C* and *D*). Furthermore, because metal responsiveness from P*_frmRA_-frmRE64H* is affected by glutathione (Fig. 8, *B* and *C*), glutathione levels were measured but found not to be significantly altered between Δ*frmR* cells expressing P*_frmRA_-frmR* or P*_frmRA_-frmRE64H* in either the presence (3.8 (±0.5) and 4.5 (±0.6) mm, respectively) or absence (4.4 (±0.8) and 3.3 (±0.4) mm, respectively) of added Zn(II).

##### Codon Optimization or De-optimization Alters FrmRE64H or FrmR Cell^−1^ but Does Not Switch Metal Perception

Loss of repression by FrmRE64H compared with FrmR could in theory be due to reduced protein abundance, for example due to impaired stability of the mutant protein. To test this suggestion, constructs were generated in which FrmRE64H codons were optimized for efficient translation (62, 63), designated P*_frmRA_-frmRE64H*^UP^. Conversely, FrmR expression was de-optimized by introduction of rare arginine codons (62, 63), designated P*_frmRA_-frmR*^DOWN^. This approach was chosen to alter abundance of the proteins while preserving the transcriptional architecture. Basal expression was enhanced in cells containing *frmR*^DOWN^ and reduced in cells containing *frmRE64H*^UP^ relative to the respective controls and yielding matched levels of basal *lacZ* expression by *frmR*^DOWN^ *versus frmRE64H*^UP^ (Fig. 9*E*). Moreover, the numbers of FrmRE64H and FrmR tetramers per cell, as determined by quantitative mass spectrometry, were indeed increased and decreased, respectively, in cells harboring the codon-altered variants (Fig. 9, *G–K*). Cells containing any of the variants, *frmRE64H*, *frmRE64H*^UP^, *frmR*, *frmR*^DOWN^, all showed enhanced expression following exposure to MNIC of formaldehyde, but crucially only the strains expressing FrmRE64H responded to Zn(II) and cobalt (Fig. 9*F*). Notably, the abundance of FrmRE64H is no less than FrmR (Fig. 9*K*), and an alternative explanation is needed for elevated basal expression in cells containing FrmRE64H.

##### ΔG_C_^Zn(II)-FrmRE64H·DNA^ Is Less than ΔG_C_^Zn(II)-FrmR·DNA^ with Apo-FrmRE64H K_DNA_ Being Weaker

**FIGURE 10.**
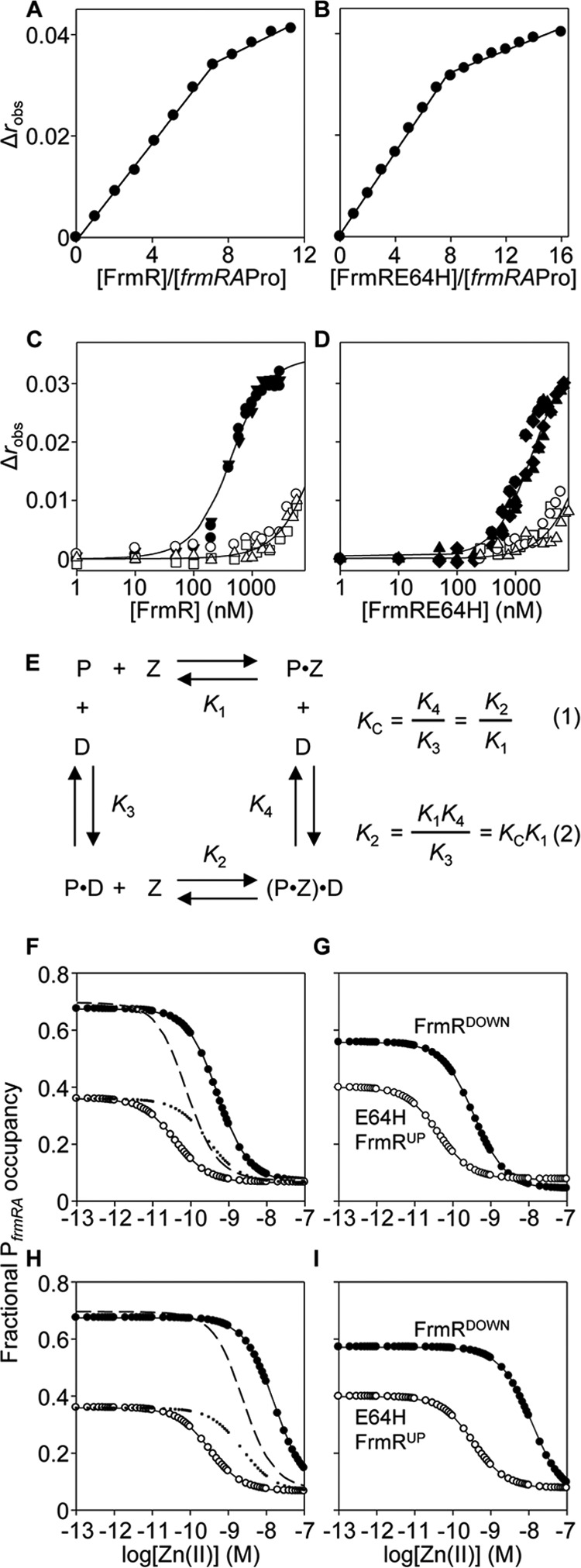
**Zn(II) weakens *K*_DNA_ of FrmR and FrmRE64H and its effect on DNA occupancy.** Anisotropy change upon titration of a high concentration of *frmRA*Pro (2.5 μm) with FrmR (*A*), FrmRE64H (*B*), or a limiting concentration of *frmRA*Pro (10 nm) (*C*) with apo-FrmR in the presence of 5 mm EDTA (*closed symbols*) or Zn(II)-FrmR in the presence of 5 μm ZnCl_2_ (*open symbols*). *D*, as *C* but using FrmRE64H. *Symbol shapes* represent individual experiments. Data were fit to a model describing a 2:1 protein tetramer (nondissociable):DNA stoichiometry (binding with equal affinity), and *lines* represent simulated curves produced from the average *K*_DNA_ determined across the experimental replicas shown. *E*, coupled thermodynamic equilibria (assuming a closed system) describing the relationship between FrmR tetramer (*P*), Zn(II) (*Z*), and P*_frmRA_* (*D*) (9, 65, 66). The coupling constant (*K*_C_) is determined from the ratio *K*_4_/*K*_3_ (*K*_DNA_^Zn(II)·FrmR^/*K*_DNA_^FrmR^) (Equation 1) and used to calculate *K*_2_ (the Zn(II) affinity of the DNA-bound protein, *K*_Zn(II)_^FrmR·DNA^) from *K*_1_ (K_Zn(II)_^FrmR^) (Equation 2). *F,* calculated fractional occupancy of P*_frmRA_* with FrmR (*filled circles*) and FrmRE64H (*open circles*) as a function of (buffered) [Zn(II)], which incorporates the determined FrmR or FrmRE64H abundance, *K*_Zn(II)_^sensor^ (off DNA), and *K*_DNA_ (Table 1). Additional lines represent hypothetical fractional occupancy of P*_frmRA_* with FrmRE64H but substituting *K*_Zn(II)_ (*dotted*) or *K*_DNA_ (*dashed*) for that of FrmR. *G,* as *F* but using the determined abundance for FrmR^DOWN^ (*solid symbols*) and FrmRE64H^UP^ (*open symbols*). *H* and *I*, as *F* and *G*, respectively, except using *K*_Zn(II)_^sensor·DNA^ (on-DNA) (calculated using the equations in *E*).

Fluorescence anisotropy was used to monitor interactions between either FrmRE64H or FrmR and a fluorescently labeled double-stranded DNA fragment of the target operator-promoter, *frmRA*Pro (Fig. 1*C*). DNA-protein stoichiometry was first determined by monitoring DNA binding to a relatively high concentration of *frmRA*Pro (2.5 μm) with saturation observed at ∼20 μm FrmRE64H or FrmR (monomer) consistent with binding of two tetramers (Fig. 10, *A* and *B*). A limiting concentration of *frmRA*Pro (10 nm) was subsequently titrated with apo- or Zn(II)-saturated FrmRE64H or FrmR in the presence of 5 mm EDTA or 5 μm Zn(II), respectively, and anisotropy data were fitted to models describing the binding of two nondissociable protein-tetramers per DNA molecule (Fig. 10, *C* and *D*). The calculated DNA binding affinities (*n* ≥ 3) are shown in Table 2. *K*_DNA_ was similarly determined for Cu(I)-FrmR (Table 2), but weak *K*_Co(II)_ precluded equivalent *K*_DNA_ estimations for Co(II)-saturated proteins (Table 1). Metal binding weakens DNA binding, but unexpectedly this is true of wild type FrmR as well as FrmRE64H.

**TABLE 2:** **DNA binding affinities and allosteric coupling free energies of FrmR and FrmRE64H** Data were determined by fluorescence anisotropy. The conditions used are as follows: 25 °C, 10 mm HEPES, pH 7.0, 60 mm NaCl, 240 mm KCl with addition of 5 mm EDTA for apoprotein titrations or 20 μm ZnCl_2_ for Zn(II)-protein titrations. Proteins were incubated with 1.2 m eq per monomer of ZnCl_2_ or CuCl for metal-loaded titrations. Δ*G*_C_ = −*RT*ln*K*_C_. NA means not applicable.

Sensor	Metal	*K*_DNA_*^a^*	Δ*G*_c_
		*m*	*kcal mol*^−*1*^
FrmR	Apo	9.94 ± 0.3 × 10^−8^	NA
	Zn(II)	3.11 ± 0.4 × 10^−6^	2.03 ± 0.08
	Cu(I)	6.54 ± 1.3 × 10^−7^	1.10 ± 0.10
FrmRE64H	Apo	4.26 ± 0.4 × 10^−7^	NA
	Zn(II)	3.51 ± 0.7 × 10^−6^	1.24 ± 0.16

*^a^* Data were fit to a model describing two nondissociable tetramers binding to *frmRA*Pro with equal affinity (*n* = 3).

The degree to which metal binding allosterically inhibits DNA binding has previously been expressed as the coupling free energy (Δ*G*_C_) calculated from the ratio of *K*_DNA_ of apo- and holo-proteins and using a standard thermodynamic function (see Refs. 9, 11, 64 and the footnotes to Table 2). This yields Zn(II)-FrmR Δ*G*_C_ = +2.03 (±0.08) kcal mol^−1^ (Δ*G*_C_^Zn(II)-FrmR·DNA^) and Zn(II)-FrmRE64H Δ*G*_C_ = +1.24 (±0.16) kcal mol^−1^ (Δ*G*_C_^Zn(II)-FrmRE64H·DNA^) (Table 2). Unexpectedly, this approach revealed that Zn(II) is less, not more, allosterically effective when binding to FrmRE64H than to FrmR, with the former having the smaller coupling free energy. However, inspection of the DNA binding curves (Fig. 10, *C* and *D*), and *K*_DNA_ values (Table 2), reveals that this results from apo-FrmRE64H having a weaker DNA affinity than apo-FrmR. These data explain the loss of basal repression by FrmRE64H. Importantly, despite a lesser Δ*G*_C_, because *K*_DNA_ of Zn(II)-FrmRE64H is not tighter than Zn(II)-FrmR (Table 2), at equivalent Zn(II) saturation DNA occupancy by FrmRE64H will still be less than FrmR, in effect rendering FrmRE64H more sensitive to de-repression. Moreover, assuming a closed system, coupled thermodynamic equilibria infer that any effect of metal binding on *K*_DNA_ is reciprocated in an effect of DNA binding on *K*_Zn(II)_ (Fig. 10*E*) (9, 61, 65, 66). Thus a smaller Δ*G*_C_^Zn(II)-FrmRE64H·DNA^ means an even tighter *K*_Zn(II)_ for DNA-bound FrmRE64H relative to FrmR. The inferred *K*_Zn(II)_^sensor·DNA^ (on-DNA) is 5.3 × 10^−9^ and 1.9 × 10^−10^ m for FrmR and FrmRE64H, respectively. A weaker *K*_DNA_ thereby contributes in two ways to the mechanism enabling metal perception by the FrmRE64H variant, and overall, a tighter *K*_Zn(II)_ plus a weaker *K*_DNA_ act in combination to confer Zn(II) sensing.

## Discussion

Substitution of one amino acid has created a metal sensor from the formaldehyde-responsive, DNA-binding transcriptional de-repressor FrmR (Fig. 2). Contrasting the biochemical properties of these two proteins (FrmR and FrmRE64H), along with endogenous *Salmonella* metal sensors (Figs. 3–7, 8*A*, and 10, *A* and *B*, and Tables 1 and 2), demonstrates what is required for metal sensing within cells. These data test (by gain-of-function) theories that have been developed from correlations between the biochemical properties of various metal sensor proteins and the metals they detect (1, 2, 6, 9, 11, 18). The single residue change in FrmRE64H tightens *K*_Zn(II)_ by ∼10-fold and weakens apo-*K*_DNA_ by ∼10-fold, and in combination these changes to metal binding and DNA binding make Zn(II) sensitivity comparable with endogenous Zn(II) sensors ZntR and Zur (Figs. 2, 8*A*, and 10, *A* and *B*). In common with recent studies of cobalt detection in other cells (6, 67), relative access (a major kinetic contribution) is invoked to explain the gain of cobalt detection by FrmRE64H, a response that is assisted by glutathione (Fig. 8).

Unexpectedly, the native FrmR protein binds Co(II), Zn(II), and Cu(I) (Fig. 3 and 4). Moreover, Zn(II) and Cu(I) are shown by fluorescence anisotropy to be allosterically effective and able to weaken *K*_DNA_, thereby raising questions about why native FrmR does not normally de-repress gene expression in response to these metal ions (Fig. 10*C* and Table 2). Crucially, by characterizing *Salmonella* ZntR and RcnR (Fig. 5, *A–E* and *J–L*), and by measuring cognate *K*_metal_ of *Salmonella* Zn(II)-sensing ZntR and Zur, Cu(I)-sensing CueR, and cobalt-sensing RcnR (Figs. 5, *F–I,* and 6, and Table 1), it becomes evident that in each case the respective metal affinity of FrmR is substantially weaker than each cognate sensor and it cannot compete (Figs. 7 and 8*A*). Values for *K*_Zn(II)_, *K*_Co(II)_, and *K*_Cu(I)_ for *Salmonella* Zur, ZntR, RcnR, and CueR determined here are comparable with analogous sensors from some other organisms (Figs. 5 and 6) (1, 11, 17, 28, 64, 68). The ability of FrmR to respond to metals *in vitro* but not within cells (Figs. 2, *A* and *E*, and 10*C*), coupled with relative *K*_metal_ values (Table 1), provides another line of evidence that metal sensing within cells is a combined product of a set of sensors (1). The best sensor in the set is the one that responds to each element (1, 11). In each case *K*_metal_ for FrmR is substantially weaker than the respective *K*_metal_ for the best in the set of sensors in *Salmonella* (Fig. 8*A*), and so it does not respond.

The E64H substitution was intended to create a metal-binding site more analogous to RcnR and indeed *K*_Co(II)_ plus *K*_Zn(II)_ and *K*_Cu(I)_ are all tighter by ∼10-fold compared with FrmR (Fig. 4 and Table 1), but they all remain weaker than the respective cognate metal sensor (Table 1 and Figs. 6 and 8*A*). Nonetheless, for Zn(II) the affinity of FrmRE64H approaches that of known Zn(II) sensors such that there is overlap in fractional metal occupancy curves as a function of [Zn(II)] (Fig. 8*A*). The free energy of coupling of Zn(II) binding to DNA binding for FrmRE64H also changes relative to FrmR (Fig. 10, *C* and *D*, and Table 2). However, the change is the opposite of what might be predicted (1, 9, 11, 64), with Zn(II) appearing to be less, not more, allosterically effective in the mutant protein (Δ*G*_C_^Zn(II)-FrmRE64H·DNA^ < Δ*G*_C_^Zn(II)-FrmR·DNA^). Importantly, these values incorporate a much weaker *K*_DNA_ for apo-FrmRE64H (Fig. 10*D*), which lowers overall promoter occupancy enhancing sensitivity to de-repression. Moreover, if regulation is dominated by metal binding to the DNA-protein complex to promote DNA dissociation, then the lesser Δ*G*_C_ of FrmRE64H infers an even tighter *K*_Zn(II)_ (assuming a closed system (9, 61, 65, 66)) of the active DNA-bound species relative to FrmR (Fig. 10*E*).

Unlike for Zn(II), the enhanced *K*_metal_ of FrmRE64H does not approach that of cognate sensors for Cu(I) or cobalt (Fig. 8*A*). Thus, relative affinity is consistent with the continued inability of FrmRE64H to detect Cu(I). However, the gain of cobalt sensing by FrmRE64H is enigmatic. In studies of the model cyanobacterium, *Synechocystis* PCC 6803, the detection of nickel and Zn(II) correlated with relative affinity and relative allostery within the set of sensors, but the detection of cobalt was attributed to relative access (1, 6, 11, 18). Somehow, the cobalt effector was preferentially available to the cobalt sensor CoaR relative to sensors for other metals. Thus, although Zn(II) sensors ZiaR and Zur had tighter affinities for Co(II) than CoaR and both were (allosterically) responsive to Co(II) *in vitro*, neither ZiaR nor Zur responded to cobalt in the cell, whereas CoaR with weaker *K*_Co(II)_ responded (6). Unlike *Synechocystis* CoaR, because FrmR has not evolved to detect cobalt, it is difficult to understand why cobalt should be channeled to FrmRE64H (Fig. 8*A* and Table 1). FrmR and cobalt-sensing RcnR do share common ancestry, and so interaction with a cobalt donor could perhaps be an evolutionary relic. Glutathione complexes are components of the buffered cellular pools for a number of metals (69). Because the substrates for formaldehyde dehydrogenase, FrmA, which is regulated by FrmR, are *S*-(hydroxymethyl)glutathione and *S*-nitrosoglutathione, it is also formally possible that FrmR can respond to glutathione adducts (33, 70). Here, we see that cobalt and Zn(II) sensing by FrmRE64H is somehow assisted by glutathione (Fig. 8, *B* and *C*). This is opposite to what has previously been observed in the detection of cellular Zn(II) in other systems where the glutathione-substitute, bacillithiol, competes with Zn(II) sensors (61), and here we see a negligible effect of glutathione on Zn(II) sensing by ZntR (Fig. 8*D*). Whether glutathione aids the detection of cobalt due to cobalt binding and trafficking or due to redox effects on the oxidation state of cobalt or its ligands remains to be established.

Basal repression by FrmRE64H is less than FrmR, and this is explained by weaker *K*_DNA_ of apo-FrmRE64H (Figs. 9 and 10, *C* and *D*). In pursuing the explanation for this phenotype, the abundance of both proteins was adjusted by optimizing or de-optimizing codons, an approach that preserves the transcriptional architecture. These changes were confirmed to increase and decrease the number of copies of FrmRE64H and FrmR per cell, respectively, with concomitant gain and loss of repression leading to matched levels of basal expression (Fig. 9, *E–K*). However, the magnitude of these changes in protein abundance alone was insufficient to switch FrmR into a metal sensor or to stop FrmRE64H from responding to Zn(II) or cobalt (Fig. 9*F*). Nonetheless, in theory, a change in relative protein abundance could alter metal competition with other sensors by mass action, and relative abundance should be added to the list of relative properties (affinity, allostery, and access) that determine which sensor is the best in the set to respond to a metal.

By how much do tighter *K*_Zn(II)_ and weaker *K*_DNA_ values of the apoprotein enhance the sensitivity of FrmRE64H to Zn(II), alone and in combination? By using the parameters set out in Tables 1 and 2, plus Fig. 9*K*, it has become possible to estimate fractional occupancy of the *frmRA* operator-promoter with repressor, either FrmR or FrmRE64H, as a function of [Zn(II)] (refer to “Experimental Procedures,” supplemental material, and Fig. 10, *F–I*). First, a weaker *K*_DNA_ of apo-FrmRE64H causes operator-promoter occupancy to be less than FrmR even in the absence of elevated Zn(II) (Fig. 10*F*), which explains the small but detectable (Figs. 2 and 9), basal de-repression. Individually, the determined tighter *K*_Zn(II)_ or weaker apo-*K*_DNA_ alone enhance the sensitivity of FrmRE64H to [Zn(II)] by ∼1 order of magnitude (*dotted* and *dashed lines* on Fig. 10*F*), although in combination they increase sensitivity by ∼2 orders of magnitude. If regulation is dominated by metal binding to (and promoting dissociation of) DNA-bound protein, then the inferred (weaker) *K*_Zn(II)_ of the DNA-adduct becomes the relevant parameter (Fig. 10*E*). Under this regime (which assumes a closed system), the weaker *K*_DNA_ of apo-FrmRE64H lessens Δ*G*_C_^Zn(II)-FrmRE64H·DNA^ and infers a tighter *K*_Zn(II)_ of DNA-bound FrmRE64H (on DNA) relative to FrmR. This, in combination with the measured tighter *K*_Zn(II)_, enhances sensitivity to [Zn(II)] by ∼3 orders of magnitude (Fig. 10*H*).

There is ambiguity about the buffered concentrations of metals in cells (1). These values are important because relative metal availability influences metal occupancy by metalloproteins (71, 72). Plausible limits on cellular buffered [Zn(II)] are defined by FrmRE64H, FrmR, FrmRE64H^UP^, and FrmR^DOWN^ (Fig. 10, *F–I*). In the absence of elevated exogenous Zn(II), for FrmRE64H to fully repress, the buffered [Zn(II)] must be held below 10^−11^ m, even if the inferred weaker (on DNA) *K*_Zn(II)_ is applied to all molecules (Fig. 10*H*). This low (sub-nanomolar) value suggests that metalloproteins acquire competitive metals such as Zn(II) when there is no hydrated metal pool. These estimates of the buffered concentration of Zn(II) are consistent with the hypothesis that metalloproteins acquire Zn(II) via associative ligand exchange from a polydisperse buffer (1), rather than a hydrated pool of ions. This represents an associative cell biology of Zn(II).

Whether or not a significant pool of hydrated ions contributes to the metallation and hence regulation of FrmRE64H (and by inference other metal sensors) remains unresolved (73). One view is that metal sensors respond to hydrated ions at ∼10^−9^ m once the buffer is saturated (73). For FrmR^DOWN^ to be unresponsive when cells are challenged with elevated exogenous Zn(II), the buffered [Zn(II)] must remain somewhere below 10^−8^ m, even if the inferred weaker (on DNA) value for *K*_Zn(II)_ is assigned to all FrmR molecules (Fig. 10*I*). This limit drops to 10^−10^ m, if the determined (off DNA) *K*_Zn(II)_ is used (Fig. 10*G*). Conversely, for FrmRE64H to respond, the buffered [Zn(II)] need only exceed 10^−11^ m using the inferred weaker (on DNA) *K*_Zn(II)_ (Fig. 10*H*). This places the intracellular [Zn(II)], at which FrmRE64H responds, somewhere within the range 10^−11^ to 10^−8^ m.

A long term aspiration is to gather analogous *K*_metal_, *K*_DNA_^apoprotein^, and *K*_DNA_^metal-protein^ values, for cognate and noncognate metals, plus protein abundance for a cells' complement of metal sensors. In this manner, comparative models of sensor occupancy with metal (as in Fig. 8*A*) could be refined to more sophisticated and comparative models of promoter occupancy by repressors, as shown in Fig. 10, *F–I*. In turn, this should render transcriptional responses to metals predictable. In closing, the (subtle) biochemical changes, which in combination enable FrmRE64H to detect a sub-set of metals, support a view that (modest) differences in the relative properties of a cells' complement of sensors dictate which sensor is the best in the set to detect each metal inside cells.

## Author Contributions

D. O. and C. P. made equivalent contributions to the conduct of the *in vitro* experiments, analysis, and preparation of the data. D. O. did the *in vivo* experiments. N. J. R. and D. O. drafted the manuscript, interpreted the significance of the data, and were responsible for the iterative design of experiments. J. C. and T. G. H. performed the quantitative LC-MS/MS. A. W. F., B. C., and D. O. developed the fractional occupancy models. N. J. R. and E. L.-L. were responsible for the conception of the program. All authors reviewed the results, edited, and approved the final version of the manuscript. N. J. R. coordinated and designed the study and provided intellectual input into all aspects of the research.

## Supplementary Material

Supplemental Data

## Footnotes

3 The abbreviations used are:

MNIC: maximum noninhibitory concentrations
TPEN: *N,N,N′,N′*-tetrakis(2-pyridylmethyl)ethylenediamine
ICP-MS: inductively coupled plasma-mass spectrometry
LMCT: ligand-to-metal charge transfer
BCS: bathocuproine sulfonate
BCA: bicinchoninic acid
BisTris: 2-[bis(2-hydroxyethyl)amino]-2-(hydroxymethyl)propane-1,3-diol.

## References

[1] Foster A. W. Osman D. Robinson N. J. (2014) Metal preferences and metallation. J. Biol. Chem. 289, 28095–28103 25160626 10.1074/jbc.R114.588145 PMC4192464

[2] Reyes-Caballero H. Campanello G. C. Giedroc D. P. (2011) Metalloregulatory proteins: metal selectivity and allosteric switching. Biophys. Chem. 156, 103–114 21511390 10.1016/j.bpc.2011.03.010 PMC3097251

[3] Irving H. Williams R. J. (1948) Order of stability of metal complexes. Nature 162, 746–747

[4] Helmann J. D. (2014) Specificity of metal sensing: iron and manganese homeostasis in *Bacillus subtilis*. J. Biol. Chem. 289, 28112–28120 25160631 10.1074/jbc.R114.587071 PMC4192466

[5] Ma Z. Faulkner M. J. Helmann J. D. (2012) Origins of specificity and cross-talk in metal ion sensing by *Bacillus subtilis* Fur. Mol. Microbiol. 86, 1144–1155 23057863 10.1111/mmi.12049 PMC3508374

[6] Patterson C. J. Pernil R. Dainty S. J. Chakrabarti B. Henry C. E. Money V. A. Foster A. W. Robinson N. J. (2013) Co(II)-detection does not follow *K*_Co(II)_ gradient: channelling in Co(II)-sensing. Metallomics 5, 352–362 23420021 10.1039/c3mt20241k

[7] Cobine P. A. George G. N. Jones C. E. Wickramasinghe W. A. Solioz M. Dameron C. T. (2002) Copper transfer from the Cu(I) chaperone, CopZ, to the repressor, Zn(II)CopY: metal coordination environments and protein interactions. Biochemistry 41, 5822–5829 11980486 10.1021/bi025515c

[8] Finney L. A. O'Halloran T. V. (2003) Transition metal speciation in the cell: insights from the chemistry of metal ion receptors. Science 300, 931–936 12738850 10.1126/science.1085049

[9] Guerra A. J. Giedroc D. P. (2012) Metal site occupancy and allosteric switching in bacterial metal sensor proteins. Arch. Biochem. Biophys. 519, 210–222 22178748 10.1016/j.abb.2011.11.021 PMC3312040

[10] McGuire A. M. Cuthbert B. J. Ma Z. Grauer-Gray K. D. Brunjes Brophy M. Spear K. A. Soonsanga S. Kliegman J. I. Griner S. L. Helmann J. D. Glasfeld A. (2013) The roles of the A and C sites in the manganese-specific activation of MntR. Biochemistry 52, 701–713 23298157 10.1021/bi301550t PMC3562352

[11] Foster A. W. Pernil R. Patterson C. J. Robinson N. J. (2014) Metal specificity of cyanobacterial nickel-responsive repressor InrS: cells maintain zinc and copper below the detection threshold for InrS. Mol. Microbiol. 92, 797–812 24666373 10.1111/mmi.12594 PMC4235346

[12] Ma Z. Cowart D. M. Scott R. A. Giedroc D. P. (2009) Molecular insights into the metal selectivity of the copper(I)-sensing repressor CsoR from *Bacillus subtilis*. Biochemistry 48, 3325–3334 19249860 10.1021/bi900115w PMC2728441

[13] Lisher J. P. Higgins K. A. Maroney M. J. Giedroc D. P. (2013) Physical characterization of the manganese-sensing pneumococcal surface antigen repressor from *Streptococcus pneumoniae*. Biochemistry 52, 7689–7701 24067066 10.1021/bi401132w PMC3859839

[14] Higgins K. A. Giedroc D. (2014) Insights into protein allostery in the CsoR/RcnR family of transcriptional repressors. Chem. Lett. 43, 20–25 24695963 10.1246/cl.130965 PMC3970791

[15] Liu T. Ramesh A. Ma Z. Ward S. K. Zhang L. George G. N. Talaat A. M. Sacchettini J. C. Giedroc D. P. (2007) CsoR is a novel *Mycobacterium tuberculosis* copper-sensing transcriptional regulator. Nat. Chem. Biol. 3, 60–68 17143269 10.1038/nchembio844

[16] Iwig J. S. Rowe J. L. Chivers P. T. (2006) Nickel homeostasis in *Escherichia coli*: the *rcnR-rcnA* efflux pathway and its linkage to NikR function. Mol. Microbiol. 62, 252–262 16956381 10.1111/j.1365-2958.2006.05369.x

[17] Iwig J. S. Leitch S. Herbst R. W. Maroney M. J. Chivers P. T. (2008) Ni(II) and Co(II) sensing by *Escherichia coli* RcnR. J. Am. Chem. Soc. 130, 7592–7606 18505253 10.1021/ja710067d PMC2435081

[18] Foster A. W. Patterson C. J. Pernil R. Hess C. R. Robinson N. J. (2012) Cytosolic Ni(II) sensor in cyanobacterium: nickel detection follows nickel affinity across four families of metal sensors. J. Biol. Chem. 287, 12142–12151 22356910 10.1074/jbc.M111.338301 PMC3320959

[19] Festa R. A. Jones M. B. Butler-Wu S. Sinsimer D. Gerads R. Bishai W. R. Peterson S. N. Darwin K. H. (2011) A novel copper-responsive regulon in *Mycobacterium tuberculosis*. Mol. Microbiol. 79, 133–148 21166899 10.1111/j.1365-2958.2010.07431.x PMC3052634

[20] Smaldone G. T. Helmann J. D. (2007) CsoR regulates the copper efflux operon *copZA* in *Bacillus subtilis*. Microbiology 153, 4123–4128 18048925 10.1099/mic.0.2007/011742-0 PMC3019219

[21] Corbett D. Schuler S. Glenn S. Andrew P. W. Cavet J. S. Roberts I. S. (2011) The combined actions of the copper-responsive repressor CsoR and copper-metallochaperone CopZ modulate CopA-mediated copper efflux in the intracellular pathogen *Listeria monocytogenes*. Mol. Microbiol. 81, 457–472 21564342 10.1111/j.1365-2958.2011.07705.x

[22] Baker J. Sengupta M. Jayaswal R. K. Morrissey J. A. (2011) The *Staphylococcus aureus* CsoR regulates both chromosomal and plasmid-encoded copper resistance mechanisms. Environ. Microbiol. 13, 2495–2507 21812885 10.1111/j.1462-2920.2011.02522.x

[23] Dwarakanath S. Chaplin A. K. Hough M. A. Rigali S. Vijgenboom E. Worrall J. A. (2012) Response to copper stress in *Streptomyces lividans* extends beyond genes under direct control of a copper-sensitive operon repressor protein (CsoR) J. Biol. Chem. 287, 17833–17847 22451651 10.1074/jbc.M112.352740 PMC3366776

[24] Sakamoto K. Agari Y. Agari K. Kuramitsu S. Shinkai A. (2010) Structural and functional characterization of the transcriptional repressor CsoR from *Thermus thermophilus* HB8. Microbiology 156, 1993–2005 20395270 10.1099/mic.0.037382-0

[25] Teramoto H. Yukawa H. Inui M. (2015) Copper homeostasis-related genes in three separate transcriptional units regulated by CsoR in *Corynebacterium glutamicum*. Appl. Microbiol. Biotechnol. 99, 3505–3517 25592736 10.1007/s00253-015-6373-z

[26] Rubio-Sanz L. Prieto R. I. Imperial J. Palacios J. M. Brito B. (2013) Functional and expression analysis of the metal-inducible *dmeRF* system from *Rhizobium leguminosarum* bv. viciae. Appl. Environ. Microbiol. 79, 6414–6422 23934501 10.1128/AEM.01954-13 PMC3811197

[27] Zhu T. Tian J. Zhang S. Wu N. Fan Y. (2011) Identification of the transcriptional regulator NcrB in the nickel resistance determinant of *Leptospirillum ferriphilum* UBK03. PLoS ONE 6, e17367 21387010 10.1371/journal.pone.0017367 PMC3046157

[28] Grossoehme N. Kehl-Fie T. E. Ma Z. Adams K. W. Cowart D. M. Scott R. A. Skaar E. P. Giedroc D. P. (2011) Control of copper resistance and inorganic sulfur metabolism by paralogous regulators in *Staphylococcus aureus*. J. Biol. Chem. 286, 13522–13531 21339296 10.1074/jbc.M111.220012 PMC3075698

[29] Luebke J. L. Shen J. Bruce K. E. Kehl-Fie T. E. Peng H. Skaar E. P. Giedroc D. P. (2014) The CsoR-like sulfurtransferase repressor (CstR) is a persulfide sensor in *Staphylococcus aureus*. Mol. Microbiol. 94, 1343–1360 25318663 10.1111/mmi.12835 PMC4264537

[30] Herring C. D. Blattner F. R. (2004) Global transcriptional effects of a suppressor tRNA and the inactivation of the regulator *frmR*. J. Bacteriol. 186, 6714–6720 15466022 10.1128/JB.186.20.6714-6720.2004 PMC522192

[31] Higgins K. A. Chivers P. T. Maroney M. J. (2012) Role of the N terminus in determining metal-specific responses in the *E. coli* Ni- and Co-responsive metalloregulator, RcnR. J. Am. Chem. Soc. 134, 7081–7093 22471551 10.1021/ja300834b PMC3375346

[32] Higgins K. A. Hu H. Q. Chivers P. T. Maroney M. J. (2013) Effects of select histidine to cysteine mutations on transcriptional regulation by *Escherichia coli* RcnR. Biochemistry 52, 84–97 23215580 10.1021/bi300886q PMC3610428

[33] Gutheil W. G. Holmquist B. Vallee B. L. (1992) Purification, characterization, and partial sequence of the glutathione-dependent formaldehyde dehydrogenase from *Escherichia coli*: a Class III alcohol dehydrogenase. Biochemistry 31, 475–481 1731906 10.1021/bi00117a025

[34] Denby K. J. Rolfe M. D. Crick E. Sanguinetti G. Poole R. K. Green J. (2015) Adaptation of anaerobic cultures of *Escherichia coli* K-12 in response to environmental trimethylamine-*N*-oxide. Environ. Microbiol. (10.1111/1462-2920.12726) PMC4949985 25471524

[35] Nobre L. S. Al-Shahrour F. Dopazo J. Saraiva L. M. (2009) Exploring the antimicrobial action of a carbon monoxide-releasing compound through whole-genome transcription profiling of *Escherichia coli*. Microbiology 155, 813–824 19246752 10.1099/mic.0.023911-0

[36] Wang S. Deng K. Zaremba S. Deng X. Lin C. Wang Q. Tortorello M. L. Zhang W. (2009) Transcriptomic response of *Escherichia coli* O157:H7 to oxidative stress. Appl. Environ. Microbiol. 75, 6110–6123 19666735 10.1128/AEM.00914-09 PMC2753066

[37] Sambrook J. Russell D. W. (2001) Molecular Cloning: A Laboratory Manual, 3rd Ed., Cold Spring Harbor Laboratory Press, Cold Spring Harbor, NY

[38] Datsenko K. A. Wanner B. L. (2000) One-step inactivation of chromosomal genes in *Escherichia coli* K-12 using PCR products. Proc. Natl. Acad. Sci. U.S.A. 97, 6640–6645 10829079 10.1073/pnas.120163297 PMC18686

[39] Simons R. W. Houman F. Kleckner N. (1987) Improved single and multicopy *lac*-based cloning vectors for protein and operon fusions. Gene 53, 85–96 3596251 10.1016/0378-1119(87)90095-3

[40] Osman D. Waldron K. J. Denton H. Taylor C. M. Grant A. J. Mastroeni P. Robinson N. J. Cavet J. S. (2010) Copper homeostasis in *Salmonella* is atypical and copper-CueP is a major periplasmic metal complex. J. Biol. Chem. 285, 25259–25268 20534583 10.1074/jbc.M110.145953 PMC2919089

[41] Osman D. Patterson C. J. Bailey K. Fisher K. Robinson N. J. Rigby S. E. Cavet J. S. (2013) The copper supply pathway to a *Salmonella* Cu,Zn-superoxide dismutase (SodCII) involves P_1B_-type ATPase copper efflux and periplasmic CueP. Mol. Microbiol. 87, 466–477 23171030 10.1111/mmi.12107

[42] Dainty S. J. Patterson C. J. Waldron K. J. Robinson N. J. (2010) Interaction between cyanobacterial copper chaperone Atx1 and zinc homeostasis. J. Biol. Inorg. Chem. 15, 77–85 19543924 10.1007/s00775-009-0555-z

[43] Kuzmic P. (1996) Program DYNAFIT for the analysis of enzyme kinetic data: application to HIV proteinase. Anal. Biochem. 237, 260–273 8660575 10.1006/abio.1996.0238

[44] Golynskiy M. V. Gunderson W. A. Hendrich M. P. Cohen S. M. (2006) Metal binding studies and EPR spectroscopy of the manganese transport regulator MntR. Biochemistry 45, 15359–15372 17176058 10.1021/bi0607406 PMC2561245

[45] Jefferson J. R. Hunt J. B. Ginsburg A. (1990) Characterization of indo-1 and quin-2 as spectroscopic probes for Zn^2+^-protein interactions. Anal. Biochem. 187, 328–336 2116741 10.1016/0003-2697(90)90465-l

[46] Simons T. J. (1993) Measurement of free Zn^2+^ ion concentration with the fluorescent probe mag-fura-2 (furaptra). J. Biochem. Biophys. Methods 27, 25–37 8409208 10.1016/0165-022x(93)90065-v

[47] Kwan C. Y. Putney J. W. Jr. (1990) Uptake and intracellular sequestration of divalent cations in resting and methacholine-stimulated mouse lacrimal acinar cells. Dissociation by Sr^2+^ and Ba^2+^ of agonist-stimulated divalent cation entry from the refilling of the agonist-sensitive intracellular pool. J. Biol. Chem. 265, 678–684 2404009

[48] Xiao Z. Wedd A. G. (2010) The challenges of determining metal-protein affinities. Nat. Prod. Rep. 27, 768–789 20379570 10.1039/b906690j

[49] Changela A. Chen K. Xue Y. Holschen J. Outten C. E. O'Halloran T. V. Mondragón A. (2003) Molecular basis of metal-ion selectivity and zeptomolar sensitivity by CueR. Science 301, 1383–1387 12958362 10.1126/science.1085950

[50] VanZile M. L. Cosper N. J. Scott R. A. Giedroc D. P. (2000) The zinc metalloregulatory protein Synechococcus PCC7942 SmtB binds a single zinc ion per monomer with high affinity in a tetrahedral coordination geometry. Biochemistry 39, 11818–11829 10995250 10.1021/bi001140o

[51] VanZile M. L. Chen X. Giedroc D. P. (2002) Structural characterization of distinct α5N and α3 metal sites in the cyanobacterial zinc sensor SmtB. Biochemistry 41, 9765–9775 12146942 10.1021/bi0201771

[52] Chang F. M. Coyne H. J. Cubillas C. Vinuesa P. Fang X. Ma Z. Ma D. Helmann J. D. García-de los Santos A. Wang Y. X. Dann C. E. 3rd Giedroc D. P. (2014) Cu(I)-mediated allosteric switching in a copper-sensing operon repressor (CsoR). J. Biol. Chem. 289, 19204–19217 24831014 10.1074/jbc.M114.556704 PMC4081955

[53] Scheller K. H. Abel T. H. Polanyi P. E. Wenk P. K. Fischer B. E. Sigel H. (1980) Metal ion/buffer interactions. Stability of binary and ternary complexes containing 2-[bis(2-hydroxyethyl)amino]-2(hydroxymethyl)-1,3-propanediol (Bistris) and adenosine 5′-triphosphate (ATP). Eur. J. Biochem. 107, 455–466 7398653

[54] Espariz M. Checa S. K. Audero M. E. Pontel L. B. Soncini F. C. (2007) Dissecting the *Salmonella* response to copper. Microbiology 153, 2989–2997 17768242 10.1099/mic.0.2007/006536-0

[55] Ammendola S. Cerasi M. Battistoni A. (2014) Deregulation of transition metals homeostasis is a key feature of cadmium toxicity in *Salmonella*. Biometals 27, 703–714 24970347 10.1007/s10534-014-9763-2

[56] Petrarca P. Ammendola S. Pasquali P. Battistoni A. (2010) The Zur-regulated ZinT protein is an auxiliary component of the high-affinity ZnuABC zinc transporter that facilitates metal recruitment during severe zinc shortage. J. Bacteriol. 192, 1553–1564 20097857 10.1128/JB.01310-09 PMC2832539

[57] Gilston B. A. Wang S. Marcus M. D. Canalizo-Hernández M. A. Swindell E. P. Xue Y. Mondragón A. O'Halloran T. V. (2014) Structural and mechanistic basis of zinc regulation across the *E. coli* Zur regulon. PLos Biol. 12, e1001987 25369000 10.1371/journal.pbio.1001987 PMC4219657

[58] Shin J. H. Jung H. J. An Y. J. Cho Y. B. Cha S. S. Roe J. H. (2011) Graded expression of zinc-responsive genes through two regulatory zinc-binding sites in Zur. Proc. Natl. Acad. Sci. U.S.A. 108, 5045–5050 21383173 10.1073/pnas.1017744108 PMC3064357

[59] Lucarelli D. Russo S. Garman E. Milano A. Meyer-Klaucke W. Pohl E. (2007) Crystal structure and function of the zinc uptake regulator FurB from *Mycobacterium tuberculosis*. J. Biol. Chem. 282, 9914–9922 17213192 10.1074/jbc.M609974200

[60] Ma Z. Gabriel S. E. Helmann J. D. (2011) Sequential binding and sensing of Zn(II) by *Bacillus subtilis* Zur. Nucleic Acids Res. 39, 9130–9138 21821657 10.1093/nar/gkr625 PMC3241647

[61] Ma Z. Chandrangsu P. Helmann T. C. Romsang A. Gaballa A. Helmann J. D. (2014) Bacillithiol is a major buffer of the labile zinc pool in *Bacillus subtilis*. Mol. Microbiol. 94, 756–770 25213752 10.1111/mmi.12794 PMC4227968

[62] Dong H. Nilsson L. Kurland C. G. (1996) Co-variation of tRNA abundance and codon usage in *Escherichia coli* at different growth rates. J. Mol. Biol. 260, 649–663 8709146 10.1006/jmbi.1996.0428

[63] Nakamura Y. Gojobori T. Ikemura T. (2000) Codon usage tabulated from international DNA sequence databases: status for the year 2000. Nucleic Acids Res. 28, 292 10592250 10.1093/nar/28.1.292 PMC102460

[64] Ma Z. Cowart D. M. Ward B. P. Arnold R. J. DiMarchi R. D. Zhang L. George G. N. Scott R. A. Giedroc D. P. (2009) Unnatural amino acid substitution as a probe of the allosteric coupling pathway in a mycobacterial Cu(I) sensor. J. Am. Chem. Soc. 131, 18044–18045 19928961 10.1021/ja908372b PMC2797707

[65] Ma Z. Jacobsen F. E. Giedroc D. P. (2009) Coordination chemistry of bacterial metal transport and sensing. Chem. Rev. 109, 4644–4681 19788177 10.1021/cr900077w PMC2783614

[66] Grossoehme N. E. Giedroc D. P. (2012) Illuminating allostery in metal sensing transcriptional regulators. Methods Mol. Biol. 875, 165–192 22573440 10.1007/978-1-61779-806-1_8

[67] Cavet J. S. Meng W. Pennella M. A. Appelhoff R. J. Giedroc D. P. Robinson N. J. (2002) A nickel-cobalt-sensing ArsR-SmtB family repressor. Contributions of cytosol and effector binding sites to metal selectivity. J. Biol. Chem. 277, 38441–38448 12163508 10.1074/jbc.M207677200

[68] Pennella M. A. Arunkumar A. I. Giedroc D. P. (2006) Individual metal ligands play distinct functional roles in the zinc sensor *Staphylococcus aureus* CzrA. J. Mol. Biol. 356, 1124–1136 16406068 10.1016/j.jmb.2005.12.019

[69] Helbig K. Bleuel C. Krauss G. J. Nies D. H. (2008) Glutathione and transition-metal homeostasis in *Escherichia coli*. J. Bacteriol. 190, 5431–5438 18539744 10.1128/JB.00271-08 PMC2493246

[70] Liu L. Hausladen A. Zeng M. Que L. Heitman J. Stamler J. S. (2001) A metabolic enzyme for *S*-nitrosothiol conserved from bacteria to humans. Nature 410, 490–494 11260719 10.1038/35068596

[71] Waldron K. J. Robinson N. J. (2009) How do bacterial cells ensure that metalloproteins get the correct metal? Nat. Rev. Microbiol. 7, 25–35 19079350 10.1038/nrmicro2057

[72] Waldron K. J. Rutherford J. C. Ford D. Robinson N. J. (2009) Metalloproteins and metal sensing. Nature 460, 823–830 19675642 10.1038/nature08300

[73] Wang D. Hosteen O. Fierke C. A. (2012) ZntR-mediated transcription of *zntA* responds to nanomolar intracellular free zinc. J. Inorg. Biochem. 111, 173–181 22459916 10.1016/j.jinorgbio.2012.02.008 PMC3408962

