# Supplementary material for: Generating a Metal-responsive Transcriptional Regulator to Test What Confers Metal Sensing in Cells
Source: J Biol Chem. 2015 Jun 24;290(32):19806–22. doi: 10.1074/jbc.M115.663427 (PMC4528141; doi:10.1074/jbc.M115.663427)
Supplement: Supplemental Data [file supp_290_32_19806__index.html]

Generating a Metal-Responsive Transcriptional Regulator to Test What Confers Metal-Sensing in Cells — Generating a Metal-responsive Transcriptional Regulator to Test What Confers Metal Sensing in Cells — Generation of a Metal Sensor — Supplemental Data 

# Generating a Metal-responsive Transcriptional Regulator to Test What Confers Metal Sensing in Cells

## Supplemental Data

- Supplemental data and Supplemental Tables S1 and S2 (.pdf, 445 KB) - Supplemental Table S1, Supplemental Table S2, Sample Dynafit script
