# Supplementary material for: Generating a Metal-responsive Transcriptional Regulator to Test What Confers Metal Sensing in Cells
Source: J Biol Chem. 2015 Jun 24;290(32):19806–22. doi: 10.1074/jbc.M115.663427 (PMC4528141; doi:10.1074/jbc.M115.663427)
Supplement: Supplemental Data [file supp_M115.663427_jbc.M115.663427-1.pdf]

Supplemental Data

Generating a Metal-Responsive Transcriptional Regulator to Test What Confers Metal-Sensing in Cells

Deenah Osman[1], Cecilia Piergentili[1], Junjun Chen[2], Buddhapriya Chakrabarti[1], Andrew W Foster[1], Elena Lurie-Luke[3], Thomas G Huggins[2], and Nigel J Robinson[1‡]

[1]*School of Biological and Biomedical Sciences and Department of Chemistry, Durham University, DH1 3LE, UK*

[2]*Procter and Gamble, Mason Business Centre, Cincinnati, OH 45040, USA*

[3]*Procter and Gamble, London Innovation Centre, Egham, TW20 9NW, UK*

Running title: *Generation of a metal-sensor*

[‡]To whom correspondence should be addressed. E-mail: nigel.robinson@durham.ac.uk

CONTENTS

TABLE S1	S-2
TABLE S2	S-3
SAMPLE DYNAFIT SCRIPT	S-3

TABLE S1. **Oligonucleotides used in this study.**

No.	Primer name	Sequence
**1**	frmR_del_F	5′-TAAACATAAAATTCTGATAGTATACCCCTATAGTATATGGAGGTCGAATGGTGTAGGCTGGAGCTGCTT-3′
**2**	frmR_del_R	5′-ATCTCTTCCTGTTACGGTGAGGGAAATTGTTATTTTAGATAAGCGCGAAGCATATGAATATCCTCCTTA-3′
**3**	gshA_del_F	5′-ATTACAGTTATGCTTATTAAAACGATTTGGACAGGCGGGAGGTCAATTTGGTGTAGGCTGGAGCTGCTT-3′
**4**	gshA_del_R	5′-CACTCCGTGAGTGGCCTTTTTCTTTTGGGTCAGGCGTGTTTTGCAAGCCACATATGAATATCCTCCTTA-3′
**5**	frmR_F	5′-GCTGAATGTGAACTGATTC-3′
**6**	frmR_R	5′-GCAAACGCCGGTATGAGTG-3′
**7**	gshA_F	5′-GACGGTTCAGGGCATGATGTG-3′
**8**	gshA_R	5′-AATGGCATTTAGTCACCTCCG-3′
**9**	PfrmRA_F	5′-GAAAGAAAGAATTCGTGTTTATTGTCTGTGGCCTG-3′
**10**	PfrmRA_R	5′-TTTTTATCGGATCCTGAATGCGGCATTCGACCTCC-3′
**11**	PfrmRA-frmR_R	5′-ACGGTGAGGGATCCTTATTTTAGATAAGCGCGAAG-3′
**12**	PfrmRA-frmRE64H_F	5′-GGTTGAAATCCATCTGAAAGATCATCTGGTCAGCGGGGAG-3′
**13**	PfrmRA-frmRE64H_R	5′-CTCCCCGCTGACCAGATGATCTTTCAGATGGATTTCAACC-3′
**14**	R17_CGC-CGA_F	5′-CCCGTGTTCGTCGAATACGCGGGCAGG-3'
**15**	R17_CGC-CGA_R	5′-CCTGCCCGCGTATTCGACGAACACGGG-3'
**16**	R19_CGC-CGA_F2	5′-GTTCGTCGAATACGAGGGCAGGTCGAAGC-3′
**17**	R19_CGC-CGA_R2	5′-GCTTCGACCTGCCCTCGTATTCGACGAAC-3′
**18**	R46_CGC-CGA_F	5′-GCCGCCGTGCGAGGCGCGTCTAATGG-3′
**19**	R46_CGC-CGA_R	5′-CCATTAGACGCGCCTCGCACGGCGGC-3′
**20**	R75_CGC-CGA_F	5′-CGCCAGATCAGCGAGCGGTTCGGATGG-3′
**21**	R75_CGC-CGA_R	5′-CCATCCGAACCGCTCGCTGATCTGGCG-3′
**22**	R87_CGC-CGA_F	5′-GAAATCGGCCATCTTCTTCGAGCTTATCTAAAATAAGGATCC-3′
**23**	R87_CGC-CGA_R	5′-GAAATCGGCCATCTTCTTCGAGCTTATCTAAAATAAGGATCC-3′
**24**	rcnR-PrcnA_F	5′-GGATAGAATTCCCGGCTATTTTATATAAGAATCCAGC-3′
**25**	rcnR-PrcnA_R	5′-AATTCGGATCCGAGAATGATTCTTAGTTGTTTTATGTGG-3′
**26**	pETfrmR_F	5′-GAACATATGCCGCATTCACCTGAAGATAAAAAAC-3′
**27**	pETfrmR_R	5′-GAAGAATTCTTATTTTAGATAAGCGCGAAGAAGATGGCC-3′
**28**	pETzur_F	5′-GGTGCCATATGGAAAAGACCACAACGC-3′
**29**	pETzur_R	5′-CTACTGGAATTCGTTTTCCGTTCAGGCTAAC-3′
**30**	pETzntR_F	5′-GAACATATGTATCGCATTGGTGAGCTGGC-3′
**31**	pETzntR_R	5′-GAAGAATTCTCAACATCCCGATTTCGCTCC-3′
**32**	pETrcnR_F	5′-GAACATATGTCACATACCATCCGGGAC-3′
**33**	pETrcnR_R	5′-GAAGAATTCCTATTTTATATAAGAATCCAGCACCTTTAATATTACG-3′
**34**	pETfrmR_E64H_SDM	5′-GAAATCCATCTGAAAGATCACCTGGTCAGCGGGGAG-3′
**35**	pETfrmR_E64H_SDM	5′-CTCCCCGCTGACCAGGTGATCTTTCAGATGGATTTC-3′
**36**	pETzntRLT2_C141Y_SDM	5′-GGAGCGAAATCGGGATATTGAGAATTCGAGCTCC-3′
**37**	pETzntRLT2_C141Y_SDM	5′-GGAGCTCGAATTCTCAATATCCCGATTTCGCTCC-3′
**38**	frmRPro_F	5′-[HEX]TTCTGATAGTATACCCCCCTATAGTATATGGAG-3′
**39**	frmRPro_R	5′-CTCCATATACTATAGGGGGGTATACTATCAGAA-3′

TABLE S2. **Sequence of codon optimised *frmRE64H*^{UP}.**

**Nucleotide and translated sequence of frmRE64H^{UP}**
atgccgcatagtccggaagataaaaaacgtattctgacccgtgttcgtcgtattcgtggt
M P H S P E D K K R I L T R V R R I R G
caggttgaagcactggaacgtgcactggaaagcggtgaaccgtgtctggcaattctgcag
Q V E A L E R A L E S G E P C L A I L Q
cagattgcagcagttcgtggtgcaagcaatggtctgatgagcgaaatggttgaaatccat
Q I A A V R G A S N G L M S E M V E I H
ctgaaagatcatctggttagcggtgaaaccacaccggatcagcgtgcagttcgtatggca
L K D H L V S G E T T P D Q R A V R M A
gaaattggtcatctgctgcgtgcatatctgaaataa
E I G H L L R A Y L K -

SAMPLE DYNAFIT SCRIPT: **Dynafit script used to determine fractional DNA occupancy with apo-FrmR.**

```
[task]
data = equilibria
task = fit

; components
; D....DNA
; P....Protein

[mechanism]
      D + P <==> DP      :    Keq1   dissociation

[constants]
      Keq1 = 9.94e-8

[concentrations]
      D = 2.49e-8

[response]
      DP = 40146667

[equilibria]
      directory C:/Data/MODELLING
      variable P
      offset = auto
      file FrmR.txt

[output]
      directory C:/Outputs

[end]
```
